# High-density binding to *Plasmodium falciparum* circumsporozoite protein repeats by inhibitory antibody elicited in mouse with human immunoglobulin repertoire

**DOI:** 10.1371/journal.ppat.1010999

**Published:** 2022-11-28

**Authors:** Iga Kucharska, Špela Binter, Rajagopal Murugan, Stephen W. Scally, Julia Ludwig, Katherine Prieto, Elaine Thai, Giulia Costa, Kan Li, Gillian Q. Horn, Yevel Flores-Garcia, Alexandre Bosch, Taylor Sicard, John L. Rubinstein, Fidel Zavala, S. Moses Dennison, Georgia D. Tomaras, Elena A. Levashina, Paul Kellam, Hedda Wardemann, Jean-Philippe Julien

**Affiliations:** 1 Program in Molecular Medicine, The Hospital for Sick Children Research Institute, Toronto, Canada; 2 Kymab Ltd., The Bennet Building (B930) Babraham Research Campus, Cambridge, United Kingdom; 3 B Cell Immunology, German Cancer Research Institute (DKFZ), Heidelberg, Germany; 4 Department of Biochemistry, University of Toronto, Toronto, Ontario, Canada; 5 Vector Biology Unit, Max Planck Institute for Infection Biology, Berlin, Germany; 6 Department of Surgery, Immunology, Molecular Genetics and Microbiology, Center for Human Systems Immunology, Duke University, Durham, North Carolina, United States of America; 7 Department of Molecular Microbiology and Immunology, Malaria Research Institute, Johns Hopkins Bloomberg School of Public Health, Baltimore, Maryland, United States of America; 8 Department of Medical Biophysics, University of Toronto, Toronto, Ontario, Canada; 9 Department of Infectious Diseases, Faculty of Medicine, Imperial College London, London, United Kingdom; 10 Department of Immunology, University of Toronto, Toronto, Ontario, Canada; University of Oxford, UNITED KINGDOM

## Abstract

Antibodies targeting the human malaria parasite *Plasmodium falciparum* circumsporozoite protein (PfCSP) can prevent infection and disease. PfCSP contains multiple central repeating NANP motifs; some of the most potent anti-infective antibodies against malaria bind to these repeats. Multiple antibodies can bind the repeating epitopes concurrently by engaging into homotypic Fab-Fab interactions, which results in the ordering of the otherwise largely disordered central repeat into a spiral. Here, we characterize *IGHV3-33/IGKV1-5*-encoded monoclonal antibody (mAb) 850 elicited by immunization of transgenic mice with human immunoglobulin loci. mAb 850 binds repeating NANP motifs with picomolar affinity, potently inhibits *Plasmodium falciparum* (Pf) *in vitro* and, when passively administered in a mouse challenge model, reduces liver burden to a similar extent as some of the most potent anti-PfCSP mAbs yet described. Like other *IGHV3-33/IGKV1-5*-encoded anti-NANP antibodies, mAb 850 primarily utilizes its HCDR3 and germline-encoded aromatic residues to recognize its core NANP motif. Biophysical and cryo-electron microscopy analyses reveal that up to 19 copies of Fab 850 can bind the PfCSP repeat simultaneously, and extensive homotypic interactions are observed between densely-packed PfCSP-bound Fabs to indirectly improve affinity to the antigen. Together, our study expands on the molecular understanding of repeat-induced homotypic interactions in the B cell response against PfCSP for potently protective mAbs against Pf infection.

## Author summary

Malaria is a life-threatening disease caused by Pf parasites transmitted by infected mosquitoes. The surface of infectious Pf sporozoites is covered by PfCSP, which contains multiple, short amino-acid repeats in its central domain. Antibodies targeting the repeats have been shown to be capable of neutralizing the infection. Here, we describe monoclonal antibody (mAb) 850, which was isolated following immunization with a PfCSP repeat-containing antigen in a transgenic mouse model modified to express human Ig heavy and light chain variable regions. mAb 850 binds PfCSP repeats with high affinity and inhibits Pf sporozoites in *in vitro* and *in vivo* models of infection. Our molecular analyses reveal that ~19 copies of mAb 850 can simultaneously bind one molecule of PfCSP and induce a spiral-like conformation of the repeat. Extensive antibody-antibody contacts between mAbs result in formation of one of the highest-density mAb-CSP complexes yet described. Our findings improve our understanding of antibody-PfCSP interactions that mediate parasite inhibition.

## Introduction

Malaria is a major global health concern, with over 400,000 deaths and 228 million cases annually, a majority of which are attributed to Pf [1]. In recent years, progress in combating the disease has halted, predominantly due to the increase in resistance of mosquito vectors to insecticides [2] and the emergence of multidrug-resistant parasites [3].

PfCSP densely covers the surface of *Plasmodium* sporozoites. It plays a critical role in parasite development in the *Anopheles* mosquito vector and establishment of infection in human liver cells [4–6]. PfCSP contains a largely disordered central region composed of only five amino acids (aa; asparagine, alanine, valine, aspartate and proline) arranged in a large number of repeating NANP and NANP-like motifs (NPDP and NVDP) [7,8]. The repeats are flanked by an N-terminal domain, containing a conserved five-amino-acid motif named Region I, and a C-terminal thrombospondin repeat (TSR) domain that anchors the protein to the sporozoite surface via a glycosylphosphatidylinositole (GPI) anchor. Unlike the N- and C-terminal domains, which harbor substantial sequence diversity, the central repeat domain displays slight variability only in its number of NANP and NVDP motifs [9,10].

On sporozoites, NANP repeats are immunodominant and antibodies against the central repeats can protect from infection in mouse models and in humans [11–16]. Interestingly, several mAbs against the PfCSP repeats exhibit homotypic Fab-Fab contacts when bound to the multiple central repeat motifs, including antibodies isolated after immunization with live Pf sporozoites under chloroquine prophylaxis (mAb 1210 [17]) and RTS,S/AS01 (mAb 311 [18,19], 239 and 399 [14]), currently the only vaccine approved against malaria. Homotypic antibody interactions were also observed in the case of murine mAbs targeting *P*. *berghei* (Pb) CSP (mAb 3D11 [20]) and *P*. *vivax* (Pv) CSP (mAb 2E10.E9 [21]). In humans, exposure to Pf sporozoites appears to drive anti-NANP antibody responses through clonal selection of high affinity germline B-cell receptors (BCRs) dominated by *IGHV3-33* and *IGKV1-5* genes [17]. Somatically acquired mutations were observed to improve Fab-Fab homotypic contacts and thus indirectly increase the apparent affinity of mAbs to the NANP repeats [14,17], but it remains unclear how frequent such homotypic contacts are observed amongst the most potent antibodies.

In this study, we characterize mAb 850 isolated from a mouse of the Kymouse platform [22] immunized with a PfCSP-based immunogen designed to elicit an antibody response capable of homotypic antibody interactions. *IGHV3-33/IGKV1-5*-encoded mAb 850 showed high affinity to PfCSP, potent Pf inhibition *in vitro* and reduction of liver parasite burden in a mouse model. Structural studies reveal that multiple copies of mAb 850 bind to the PfCSP repeats concurrently, and form some of the most extensive Fab-Fab interactions yet described. These molecular insights into PfCSP repeat binding improve our understanding of antibody recognition as linked to Pf inhibition.

## Results

### Immunogen design to evaluate antibody responses in mice of the Kymouse platform

To develop an immunogen that promotes the activation of NANP-reactive B cells whose BCRs can engage in homotypic Fab-Fab interactions, we designed a construct in which the heavy chain (HC) of the 1210 Fab, which is capable of forming strong homotypic interactions [17], is N-terminally linked to 5.5 NANP repeat motifs via an 8-aa linker. As designed, approximately half of the NANP repeats would be bound in a pre-formed NANP-1210 Fab complex upon co-transfection with the 1210 Fab kappa chain (KC), while the other half of the NANP repeats would remain accessible for BCR engagement.

**Fig 1:**
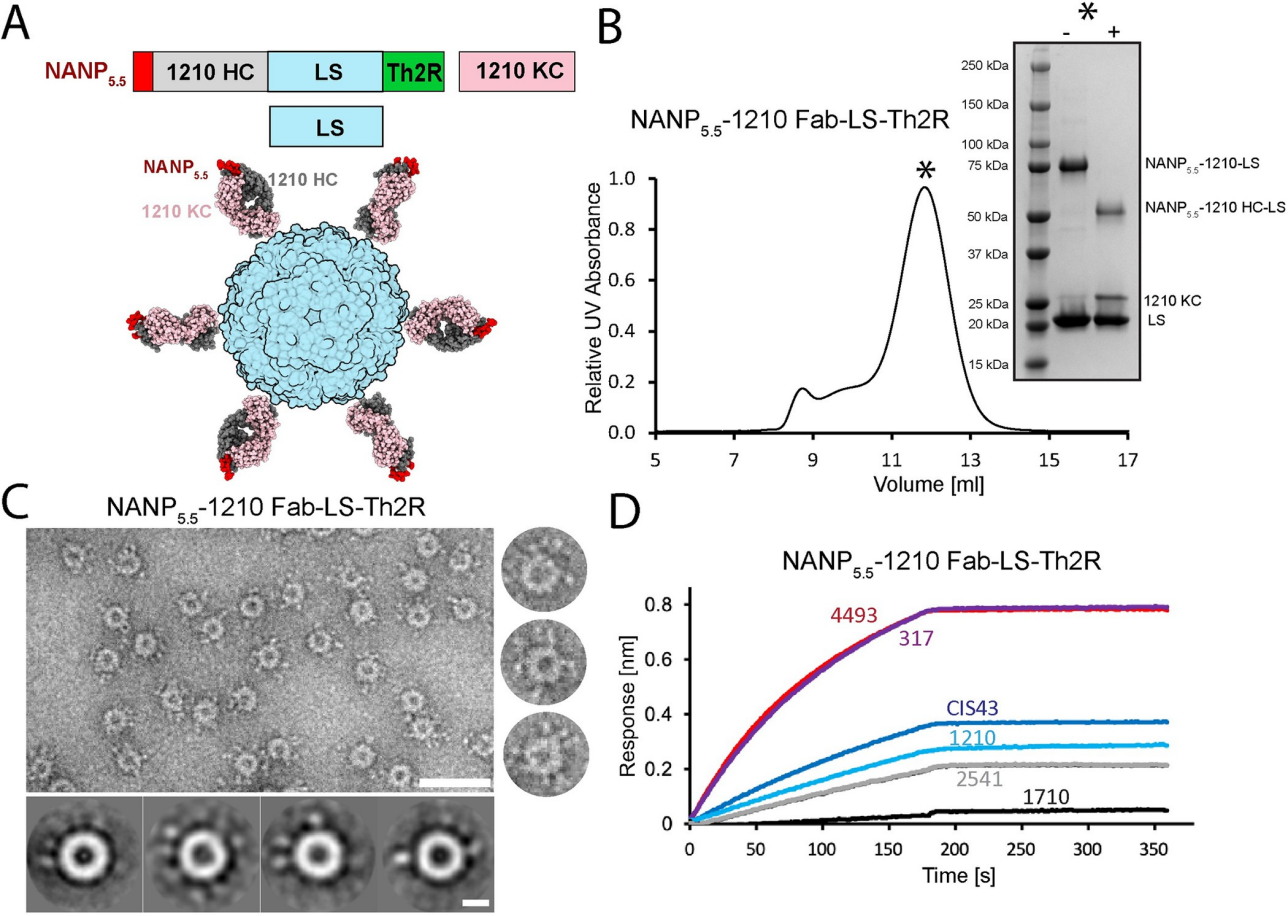
Design and characterization of NANP_5.5_-1210 Fab-LS-Th2R nanoparticles. (**A**) Schematic representation of NANP_5.5_-1210 Fab-LS-Th2R nanoparticle construct design: NANP_5.5_ peptide (red), 1210 Fab HC (grey), 1210 Fab KC (pink), LS (blue), Th2R (green). (**B**) Size exclusion chromatogram and SDS-PAGE analysis confirm the expected size of the NANP_5.5_-1210 Fab-LS-Th2R nanoparticles. The main fraction containing NANP_5.5_-1210 Fab-LS-Th2R nanoparticles is highlighted with a star, while reducing and non-reducing conditions are indicated by + and -, respectively. (**C**) Top left panel—negative stain EM image of NANP_5.5_-1210 Fab-LS-Th2R nanoparticles show the correct assembly of the purified nanoparticles. Scale bar– 50 nm. Right panels–representative individual NANP_5.5_-1210 Fab-LS-Th2R nanoparticles. Bottom panels—selected 2D class averages of NANP_5.5_-1210 Fab-LS-Th2R nanoparticles. Scale bar– 10 nm. (**D**) NANP_5.5_-1210 Fab-LS nanoparticles associate with Fabs against PfCSP repeat region (Fabs 4493 [12], 317 [18], CIS43 [27], 1210 [17], 2541 [12]) but not with a Fab against the C-terminal domain of CSP (Fab 1710 [28]) as measured by BLI. Data shown are representative of three independent measurements.

To improve immunogenicity, we fused the 1210 HC construct to the *Aquifex aeolicus* lumazine synthase (LS) monomer, leveraging its self-assembling properties to form a 60-mer nanocage displaying the antigen-Fab complex on its surface [23]. LS has shown promise as a scaffold for nanoparticle immunogens against several different pathogens, including HIV-1 [24] and SARS-CoV-2 [25]. The construct also included the Th2R epitope of PfCSP [26] linked to the C-terminus of LS to provide T cell help (**Fig 1A**).

The correct assembly of NANP_5.5_−1210 Fab-LS-Th2R nanoparticles was confirmed by gel filtration chromatography (**Fig 1B**) and negative-stain electron microscopy (EM, **Fig 1C**), which revealed that the nanocages had an average diameter of ~15 nm, consistent with the 60-meric assembly [23]. In biolayer interferometry (BLI) experiments, the unbound NANP portion of NANP_5.5_-1210 Fab-LS-Th2R nanoparticles was efficiently recognized by NANP-binding mAbs (4493 [12], 317 [18], CIS43 [27], 1210 [17], 2541 [12]), confirming it is accessible and can be engaged by a range of antibodies with distinct binding characteristics, but not by an antibody specific to the PfCSP C-terminus (1710 [28]) (**Fig 1D**).

The antibody response elicited by this immunogen was evaluated in the Kymouse platform that possesses human Ig V(D)J genes [22]. To compare the response induced by these NANP_5.5_-1210 Fab-LS-Th2R nanoparticles to that of conventional immunogens, we also immunized mice from the Kymouse platform with two nanoparticles where the NANP_5.5_ epitope was unconstrained in the context of LS (NANP_5.5_-LS-Th2R) or lower valency (24-mer) ferritin nanocages (NANP_5.5_-ferritin-TSR), as well as full-length recombinant PfCSP. Each immunogen group induced similarly strong serum antibody titers against the NANP repeats after the boost immunization (**S1A Fig**). Moreover, the antibody response elicited by each immunogen displayed strong and comparable level of functional activity as measured by the inhibitory capacity of post-immune sera against Pf traversal of hepatocytes *in vitro* (**S1B Fig**).

### mAb 850 binds the NANP repeat with high affinity and achieves potent parasite inhibition

**Fig 2:**
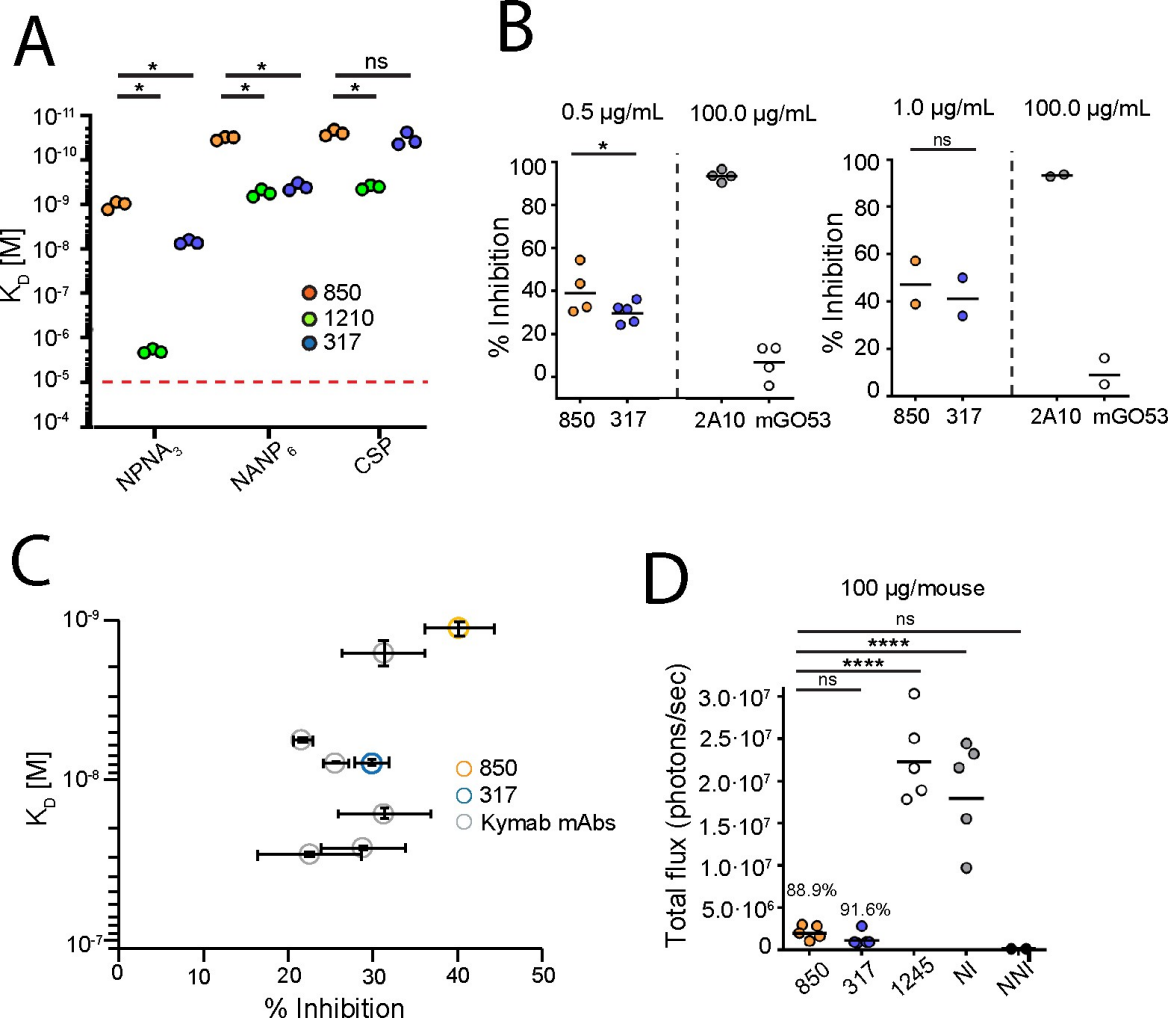
Parasite inhibition and binding of mAb 850. (**A**) Affinity of mAb 850 in comparison to mAbs 1210 and 317 as measured by SPR. (**B**) Capacity of mAb 850 to inhibit the hepatocyte traversal activity of Pf sporozoites *in vitro*, as compared to mAb 317 [18]. mAbs 850 and 317 were tested at 0.5 μg/mL (left panel) and 1.0 μg/mL (right panel), while humanized versions of murine mAbs 2A10 [32] and mGO53 [43] were used at 100 μg/mL as technical positive and negative controls, respectively. (**C**) mAb 850 displayed the highest affinity to NANP repeats and highest inhibition of Pf sporozoites at 0.5 μg/mL among isolated Kymab antibodies and mAb 317. Affinity and inhibition values are displayed as geometric and arithmetic means, respectively, while error bars represent standard error of the mean (SEM) (**see also S3 Fig**). (**D**) Assessment of antibody *in vivo* protection. Parasite liver burden load was measured by bioluminescence of Pb sporozoites expressing luciferase-conjugated PfCSP after passive transfer of 100 μg of antibody in mice (N = 5). Numbers above the data points indicate the percent inhibition of the mean parasite burden relative to that of naïve infected (NI) control mice (i.e., % inhibition). Inhibition of mAb 850 was compared to mAb 317 and negative control mAb 1245. NNI—naïve non-infected control mice. Red dashed line in **A** denotes limit of detection. Black horizontal lines in **B**–**C** indicate geometric means. P values were calculated by Bonferroni multiple-comparisons test (**B** and **C**) and Mann–Whitney test (**A**). *P < 0.05; ****P < 0.0001; n.s., statistically non-significant differences.

To further characterize the antibodies elicited by the NANP_5.5_-1210 Fab-LS-Th2R and comparator immunogens, we isolated single germinal center B cells and plasma cells from mice of the Kymouse platform at day 7 after the last of three immunizations. After RT-PCR-based paired Ig gene amplification, selected monoclonal antibodies were recombinantly expressed in HEK293 cells and screened for binding to PfCSP by ELISA (**S2 Fig**). Antibodies displaying detectable binding (**S2A Fig, S1 Table**) were further assessed using surface plasmon resonance (SPR) to determine their binding profiles to recombinant full-length PfCSP and to peptides containing NANP motifs of different lengths (NANP_6_ and NPNA_3_) (**Figs 2A,** **S3A** and **S4**). mAb 850 recognized these PfCSP-derived peptides with the highest affinity of the Kymab antibodies that we characterized, and with higher affinity compared to mAbs 317 and 1210. Affinity of mAb 850 to recombinant full-length PfCSP was comparable to mAb 317, but significantly higher than mAb 1210, making this mAb one of the highest-affinity NANP binders yet described, with a K_D_ of 1.0 X 10^−9^ M, 2.5 X 10^−11^ M and 3.2 X 10^−11^ M to the NPNA_3_ and NANP_6_ peptides, and recombinant full-length PfCSP, respectively. Interestingly, mAb 850 also appeared to be more cross-reactive than mAbs 1210 and 317, with higher affinity to N-terminal peptides KQPADGNPDPNANPN, NPDPNANPNVDPNANP and NVDPNANPNVDPNANPNVDP (**S4** and **S5 Figs**).

mAb 850 displayed the highest inhibition of Pf traversal of hepatocytes *in vitro* at 0.5 μg/mL (40.2%), compared to other Kymab *IGHV3-33/IGKV1-5-*encoded antibodies and potent mAb 317 (28.4%) (**Figs 2B and S3B** and S**3C**). The combination of high affinity to NPNA_3_ peptide with potent inhibition of Pf traversal of hepatocytes at 0.5 μg/mL rendered mAb 850 noteworthy amongst the isolated Kymab antibodies (**Fig 2C**). To confirm the inhibitory function of mAb 850 *in vivo* and support its further investigation, mice were infected intravenously with 2000 PfCSP-expressing transgenic Pb parasites [29] after passive transfer of 100 μg of antibody. mAb 850 showed a high level of liver burden inhibition (88.9%), similar to that observed for mAb 317 (91.6%) (**Fig 2D**).

### mAb 850 recognizes its core NANP epitope similarly to human *IGHV3-33/IGKV1-5* antibodies

**Fig 3:**
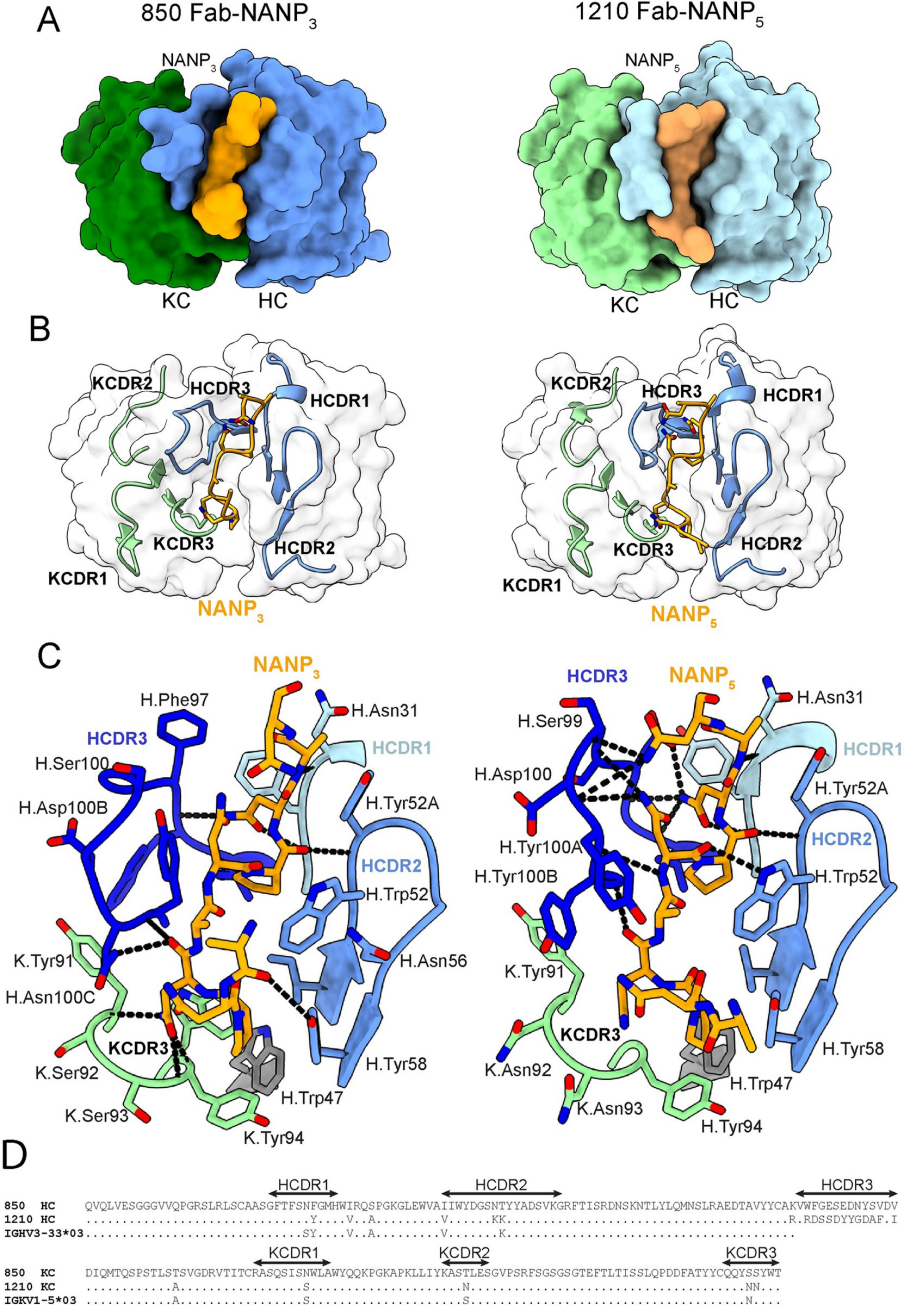
Molecular basis of NANP peptide recognition by mAb 850. (**A**) Top views of the NANP peptides (orange) in the binding groove of the 850 Fab (left panel) and 1210 Fab (right panel) shown as surface representation (HCs shown in blue and KCs shown in green). (**B**) Conformations of HC and KC CDR regions (shown in blue and green, respectively) of Fabs 850 (left panel) and 1210 (right panel) in Fab-NANP peptide (depicted in orange) co-crystal structures. (**C**) Interactions between 850 Fab and peptide NANP_3_ (left panel) and 1210 Fab and peptide NANP_5_ (right panel). H-bonds are shown as black dashes, NANP peptides are shown in orange, HCDR1, HCDR2, HCDR3 and KCDR3 residues are shown in navy, blue, light blue and green, respectively. Fab residues are annotated with H or K letters to indicate heavy and kappa light chain, respectively. (**D**) Amino acid sequence alignment of mAbs 850 and 1210 with the germline *VH* (top) and *VK* (bottom) gene segments.

To understand the molecular basis underlying the Pf inhibitory activity of mAb 850, we obtained a co-crystal structure of 850 Fab in complex with the NANP_3_ peptide at 2.2 Å resolution, as well as a crystal structure of unliganded 850 Fab at 2.0 Å (**Figs 3 and S6 and Table 1**). No significant changes in conformation for mAb 850 were observed upon binding the NANP_3_ peptide (all-atom RMSD between the two structures equals 0.6 Å, **S7A Fig**), with the exception of the orientation of side-chains of Heavy Chain Complementarity-Determining Region 3 (HCDR3) residues Asn100C and Tyr100D (**S6A–S6B** and **S7B Figs**). mAb 850 recognizes the NANP_3_ peptide in an extended conformation with residues number three to six (NPNA motif) adopting a type I β-turn (**Fig 3**). Overall, the 850 Fab-NANP_3_ crystal structure and the previously-described 1210 Fab-NANP_5_ crystal structure (PDB ID: 6D01 [17];) are remarkably similar (backbone RMSD between the Fab variable domains equals 1.3 Å; backbone RMSD between the peptides equals 1.5 Å, **S7C Fig**), with differences mainly in the N- and C-termini of the peptide, and HCDR3 regions (**Fig 3C** and **3D**). Indeed, in the 850 Fab-NANP_3_ co-crystal structure, HCDR3 residues are positioned away from the peptide and do not form as extensive a H-bonding network as between the mAb 1210 HCDR3 and the NANP_5_ peptide (**Fig 3C**). On the other hand, in the 850 Fab-NANP_3_ crystal structure, H-bonds are present between the peptide’s N_5_ and the Kappa Chain Complementarity-Determining Region 3 (KCDR3), which are absent in the 1210 Fab-NANP_5_ crystal structure (**Fig 3C**). Similar to mAb 1210, mAb 850 contains a germline-encoded Trp residue at position 52 of the HCDR2 (**Fig 3D**). H.Trp52 has previously been shown to be a key determinant of PfCSP repeat affinity in *IGHV3-33/IGKV1-5-*encoded antibodies [12,13,17,18]; in the 850 Fab-NANP_3_ crystal structure, this residue contributes 78 Å^2^ of buried surface area (BSA) and forms van der Waals interactions with N_7_ and P_8_ residues of the peptide (**Fig 3C**). mAb 850 contains 7 residues in the HC and 4 in the KC that are somatically hypermutated (SHM), and four of these residues contact the peptide directly (H.Asn31, H.Phe32 and H.Ile50 and K.Ser92; **Figs 3D** and **S8**).

**Table 1:** X-ray crystallography data collection and refinement statistics.

	850 Fab	850 Fab-NANP_3_
Beamline	APS 23-ID-D	APS 23-ID-D
Wavelength (Å)	1.033167	1.033167
Space group	I 4_1_	I 4_1_
Cell dimensions	137.2, 137.2, 63.8	137.5, 137.5, 63.6
α, β, γ (°)	90.0, 90.0, 90.0	90.0, 90.0, 90.0
Resolution (Å)*	34.29–2.00 (2.10–2.00)	28.86–2.20 (2.30–2.20)
No. molecules in ASU	1	1
No. observations	318,774 (43,412)	232,193 (16,069)
No. unique observations	40,192 (5,426)	30,049 (3,487)
Multiplicity	7.9 (8.0)	7.7 (4.5)
R_merge_ (%)^†^	15.8 (64.1)	14.7 (66.8)
R_pim_ (%)^‡^	5.8 (23.9)	5.5 (34.8)
<I/**σ** I>	8.6 (2.0)	10.6 (1.9)
CC_1/2_	0.995 (0.706)	0.996 (0.484)
Completeness (%)	100.0 (100.0)	99.6 (97.3)
***Refinement Statistics***		
Reflections used in refinement	40,173 (5,426)	30,038 (3,487)
Reflections used for R-free	1,991 (269)	1,506 (175)
Non-hydrogen atoms	4,708	4,475
Macromolecule	4,296	4,319
Water	388	156
R^§^_work_/ R^¶^_free_	0.174/ 0.212	0.169/ 0.216
***Rms deviations from ideality***		
Bond lengths (Å)	0.004	0.003
Bond angle (°)	0.71	0.64
***Ramachandran plot***		
Favored regions (%)	98.0	97.5
Allowed regions (%)	2.0	2.5
***B-factors (Å*** ^***2***^ ***)***		
Wilson B-value	23.0	34.8
Average B-factors	28.8	42.0
Average macromolecule	28.3	42.0
Average heteroatom	-	-
Average water molecule	33.9	42.4

* Values in parentheses refer to the highest resolution bin.

† R_merge_ = ∑hkl ∑i | Ihkl, i—< Ihkl > | / ∑hkl < Ihkl >.

‡ R_pim_ = ∑hkl [1/(N– 1)]1/2 ∑i | Ihkl, i—< Ihkl > | / ∑hkl < Ihkl >.

§ R_work_ = (∑ | |Fo |—|Fc | |) / (∑ | |Fo |)

¶ 5% of data were used for the R_free_ calculation.

### High-affinity, high-density 850 Fab-PfCSP interaction

**Fig 4:**
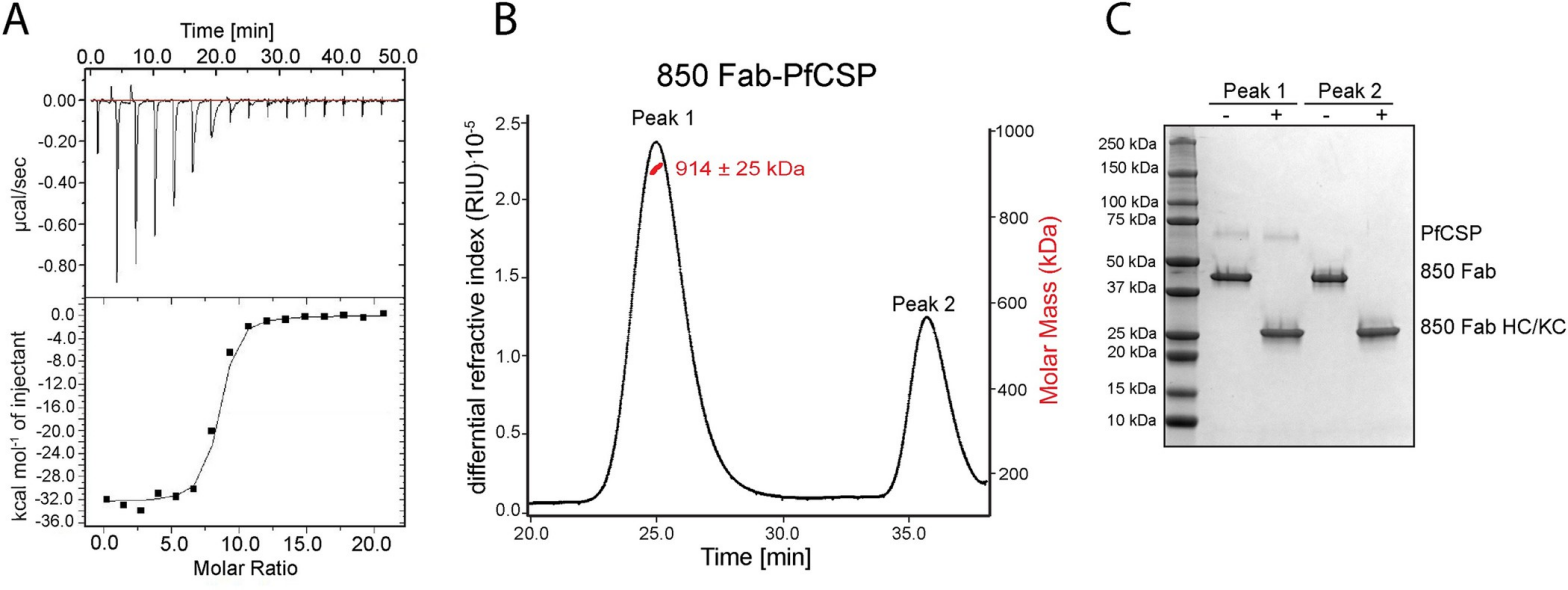
Binding of 850 Fab to full-length recombinant PfCSP. (**A**) ITC analysis of 850 Fab binding to PfCSP at 25°C. Upper panel—raw data of PfCSP (13 μM) in the sample cell titrated with 850 Fab (1340 μM) in the syringe, bottom panel—plot and trendline of heat of injectant corresponding to the raw data. (**B**) Results from SEC-MALS for the 850 Fab-PfCSP sample, where the 850 Fab is in molar excess. Measurement of the molar mass of the eluting complex is shown as a red line. Mean molar mass and standard deviation are as indicated. (**C**) SDS-PAGE analysis of resulting Peaks 1 and 2 for the 850 Fab-PfCSP sample from SEC-MALS. Each peak was sampled in reducing and non-reducing conditions as indicated by + and -, respectively.

To further characterize the binding of 850 Fab to recombinant full-length PfCSP, we performed isothermal titration calorimetry (ITC). The solution-based binding confirmed a high-affinity interaction, with a K_D_ value of 6.1 ± 1.6 X 10^−8^ M (**Fig 4A**). The stoichiometry is characteristic of several Fabs binding to a single PfCSP molecule, although the exact number of Fabs is challenging to establish using this technique provided the difficulty in accurately determining the protein concentration of PfCSP [21]. As such, we performed size exclusion chromatography coupled with multi-angle light scattering (SEC-MALS) to uncover the molecular weight of the Fab-PfCSP complex in solution. The eluting complex was determined to be 914 ± 25 kDa, which corresponds to ~18–19 copies of 850 Fab bound to one PfCSP molecule (**Fig 4B–4C**). Thus, the 850 Fab-PfCSP complex contains approximately seven Fabs more than the 1210 Fab-PfCSP complex measured with the same technique [17].

### mAb 850 homotypic contacts revealed by cryoEM

**Fig 5:**
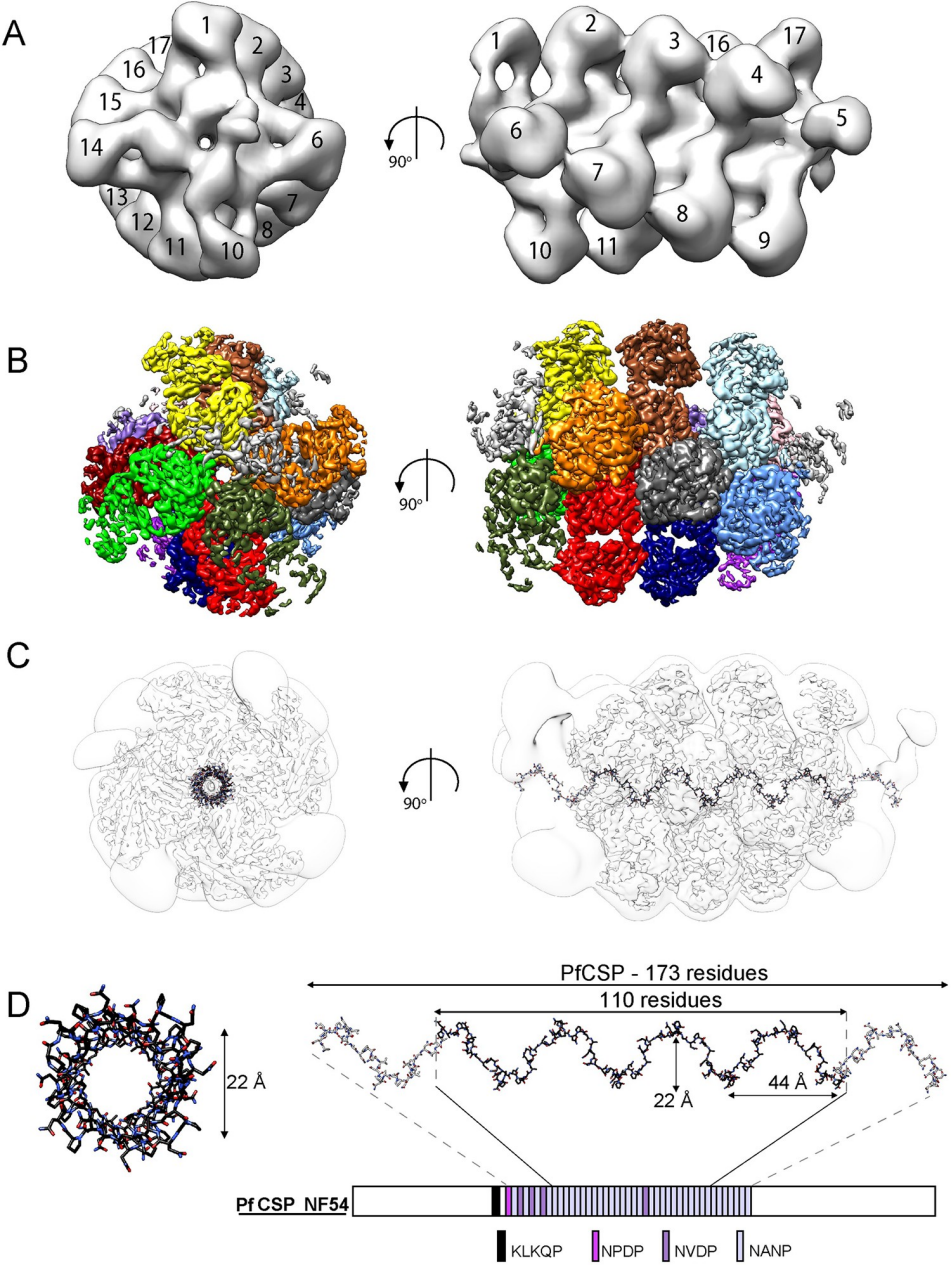
Spiral organization of the 850 Fab-PfCSP complex. (**A**) Low-pass filtered (20 Å) cryoEM map of the 850 Fab-PfCSP complex with observable 850 Fabs numbered. **(B)** Side and top view of the cryoEM map of the 850 Fab-PfCSP complex with densities corresponding to individual Fabs colored in different shades of green, red and blue. (**C**) 3.4 Å and low-pass filtered (20 Å) cryoEM maps of the 850 Fab-PfCSP complex are shown as transparent gray and white surfaces, respectively. The PfCSP model built into the cryoEM map is depicted in black, while PfCSP model extension predicted to localize in the lower resolution region of the cryoEM map is shown in grey. **(D)** The PfCSP model built into the cryoEM map corresponds to 110 residues of the PfCSP central repeat domain and is shown in black as sticks. PfCSP residues predicted to be localized in the lower resolution parts of the 850 Fab-PfCSP are depicted in grey. Models are aligned to the schematic representation of the PfCSP protein sequence.

To characterize possible homotypic interactions between 850 Fabs when bound to PfCSP, we performed cryo-electron microscopy (cryoEM) analysis of the SEC-purified 850 Fab-PfCSP complex (**S9 Fig**). A 3.4 Å resolution map of the complex was obtained (**Figs 5**, **S9** and **S10** **and Table 2**). While visualization of the map low-pass filtered to 20 Å resolution shows that the 850 Fab-PfCSP complex contains ~19 copies of 850 Fab (**Fig 5A**), consistent with the SEC-MALS characterization, flexibility at the PfCSP C- and N- termini (**S1 Video**) resulted in density too weak to warrant modelling of the five peripheral Fabs in the high-resolution structure (**Fig 5B**). The angle between adjacent 850 Fabs is ~95°, with approximately four Fabs bound per one turn of the spiral (**Fig 5A–5B**). Our modelling suggests that the PfCSP spiral comprising the peripheral regions of the low-pass filtered map would consist of ~170 residues, and thus, would accommodate all central repeats, including N-terminal NVDP repeats (**Fig 5D**). 110 of the ~170 residues in the PfCSP central region are well-resolved in the high-resolution structure and are located in the core of the complex, forming a spiral of 44 Å pitch and 22 Å diameter (**Fig 5C–5D**). Even though local resolution in the core of the map is ~2.8 Å (**S10E Fig**), we were unable to determine which part of the core density corresponded to NVDP repeats, and therefore assigned the density to the high-affinity NANP repeats. The conformations of the 850 Fab and the core PfCSP peptide are highly similar between the cryoEM and crystal structures (all-atom RMSD between the Fab variable domains: 0.4 Å; backbone RMSD between the NANP repeats: 1.7 Å, **S11 Fig**), with differences only at the extremities of the NANP repeat peptide.

**Table 2:** CryoEM data collection and refinement statistics.

***Data Collection***	
Electron microscope	Titan Krios G3
Camera	Falcon 4
Voltage (kV)	300
Nominal magnification	75,000
Calibrated physical pixel size (Å)	1.03
Total exposure (e- /Å^2^)	45
Number of frames	30
***Image Processing***	
Motion correction software	cryoSPARCv2
CTF estimation software	cryoSPARCv2
Particle selection software	cryoSPARCv2
3D map classification and refinement software	cryoSPARCv2
Micrographs used	4306
Particles selected	1,295,064
Global resolution (Å)	3.4
Particles contributing to final maps	168,235
***Model Building***	
Modeling software	Coot, phenix.real_space_refine
Number of residues built	6,215
RMS (bonds)	0.008
RMS (angles)	0.608
Ramachandran outliers (%)	0
Ramachandran allowed (%)	2.4
Ramachandran favoured (%)	97.6
Rotamer outliers (%)	0.1
Clashscore	2.6
MolProbity score	1.1
EMRinger Score	2.1

**Fig 6:**
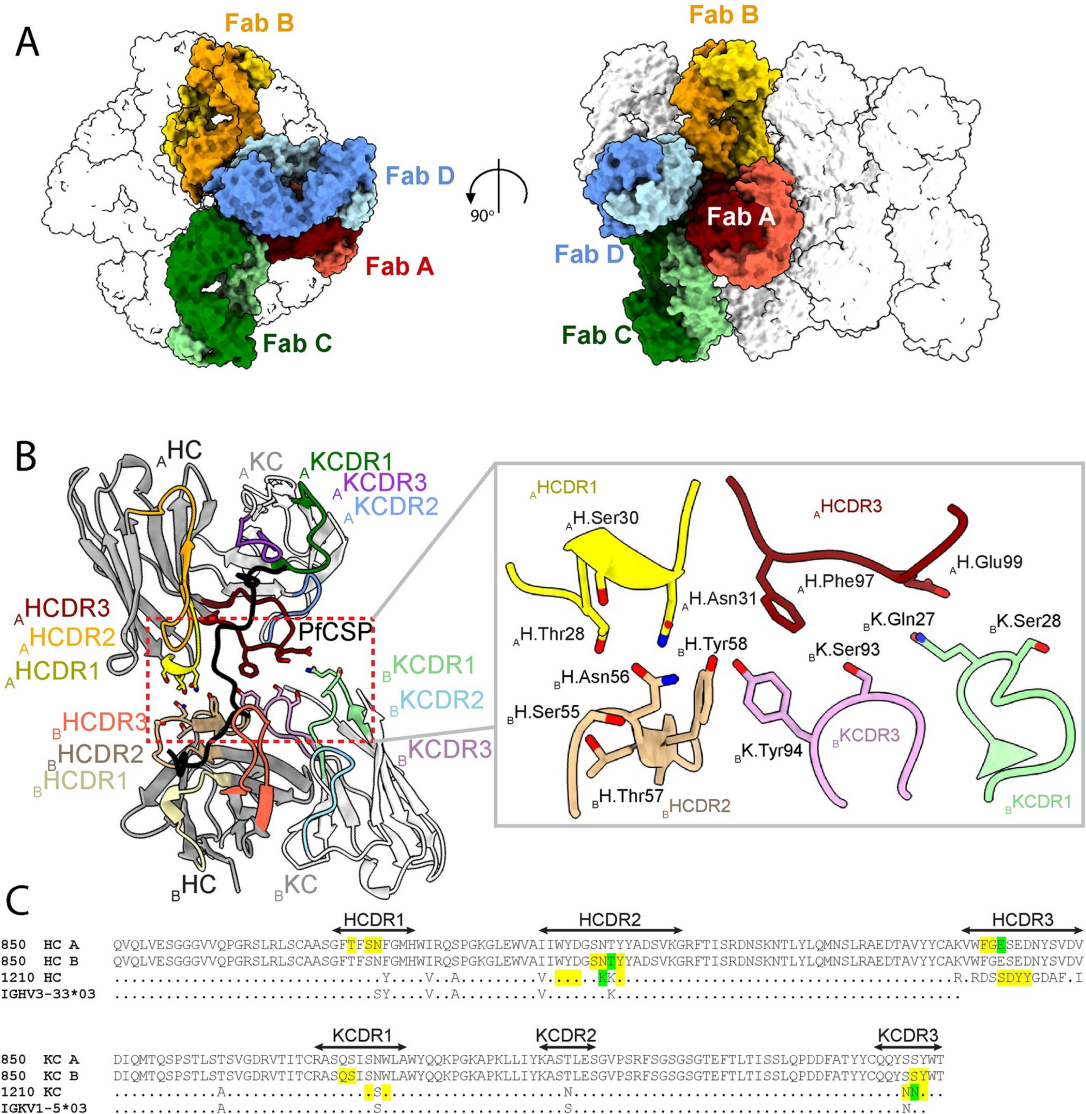
Homotypic contacts formed by adjacent 850 Fabs in 850 Fab-PfCSP cryoEM complex. (**A**) Surface representation of the 850 Fab-PfCSP model with Fab A highlighted in red, and Fabs forming homotypic contacts with Fab A highlighted in yellow (Fab B), green (Fab C) and blue (Fab D). HCs and KCs are depicted with darker and lighter shades, respectively. (**B**) Interface between adjacent Fabs A and B consists of HCDR1 and HCDR3 of Fab A and KCDR1 and 3, and HCDR2 of Fab B. The HC and KC of 850 Fab are colored dark grey and white, respectively. HCDR1, 2, and 3 of Fab A are colored dark red, orange and yellow, while the HCDR1, 2, and 3 of Fab B are colored coral, dark brown and light brown. KCDR1, 2, and 3 of Fab A are colored green, blue and purple, while the KCDR1, 2, and 3 of Fab B are colored light green, light blue and pink. PfCSP is shown in black. Residues forming Fab-Fab contacts are labeled with the position of the Fab (A and B) indicated in subscript. (**C**) Amino acid sequence alignment of mAbs 850 and 1210 with the germline *VH* (top) and *VK* (bottom) Ig gene segments. Yellow highlight: germline-encoded residues involved in homotypic interactions; green highlight: somatically hypermutated residues involved in homotypic interactions that do not form interactions with PfCSP.

The PfCSP epitope for a single Fab can be defined by 11 aa (ANPNANPNANP), with the three C-terminal residues constituting the beginning of the epitope for the adjacent Fab. When considering two consecutive Fabs as a single binding unit, the BSA of the Fabs is 1153 Å^2^, and 1398 Å^2^ for PfCSP. Adjacent 850 Fabs in the cryoEM model form contacts with each other through three distinct interfaces (**Figs 6** and **S12**). The interface between Fab A and B has 375 Å^2^ of BSA, involves HCDR1 and 3 of Fab A and HCDR2, KCDR1 and 3 of Fab B (**Fig 6B**). Two somatically hypermutated residues of Fab 850 are involved in homotypic contacts without any contacts to PfCSP: H.Thr57 and H.Glu99, with H.Thr57 forming Fab-Fab interactions through backbone atoms only and its sidechain pointing away from the adjacent Fab (**Fig 6B–6C**). The contacts contributing to other interfaces (Fab A-C and Fab A-D, **S12 Fig**) are mainly through residues in framework regions (FR) of adjacent Fabs. Thus, mAb 850 displays both classical and homotypical affinity maturation.

To investigate the role of affinity maturation in enhancing Fab-Fab contacts, somatically mutated HC residues H.Thr57 and H.Glu99 were reverted to their inferred germline precursors (Thr57Lys and Glu99Ser: subsequently named H-57/99). We performed ITC studies to evaluate binding of wild-type (WT) and H-57/99 germline-reverted mutant 850 Fabs to NANP_3_ and NANP_5_ peptides (**S13 Fig**). NANP_3_ can bind one Fab, while NANP_5_ is of sufficient length to accommodate two Fabs binding simultaneously, and therefore allows homotypic interactions to occur. As expected based on the structure, ITC studies with the NANP_3_ peptide revealed that the binding affinities of the WT 850 Fab and H-57/99 germline-reverted 850 Fab were nearly identical (K_D_’s of 1.7 ± 0.6 and 4.2 ± 0.5 X 10^−9^ M, respectively). On the other hand, the WT 850 Fab had slightly higher binding affinity (K_D_ = 9.8 ± 4.4 X 10^−9^ M) to NANP_5_ where homotypic interactions are possible, compared to the H-57/99 germline-reverted mutant 850 Fab (K_D_ = 28 ± 1.9 X 10^−9^ M). These findings confirm our structural observation that homotypic 850 Fab-Fab contacts are largely mediated through germline-encoded residues that confer high affinity binding to the PfCSP NANP repeat, and that affinity maturation at positions H-57 and H-99 played only a minor role in strengthening the homotypic contacts for mAb 850.

**Fig 7:**
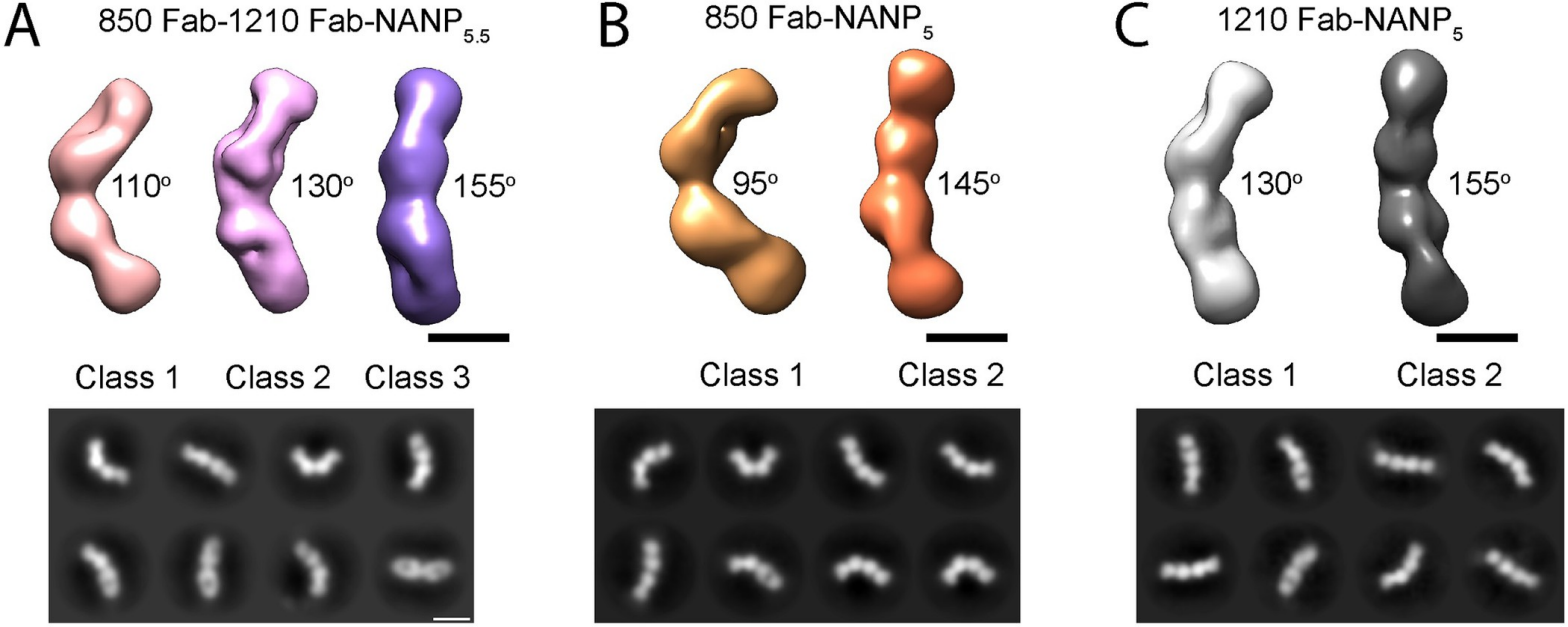
Orientation of the Fab 850- and Fab 1210-NANP repeat peptide complex. Negative stain electron microscopy 3D classes (upper panels) and representative 2D class averages (bottom panels) of **A**) 850 Fab-1210 Fab-NANP_5.5_, (**B**) 850 Fab-NANP_5_, and (**C**) 1210 Fab-NANP_5_ complexes. The approximate angle between adjacent Fabs in each class is indicated. Scale bars on 3D and 2D classes: 50 nm.

To understand how the NANP_5.5_-1210-Fab immunogen led to the elicitation of mAb 850, we performed negative stain EM of the NANP_5.5_-1210-Fab-850 Fab co-complex and compared it with the 850 Fab-NANP_5_ and 1210 Fab-NANP_5_ [21] complexes (**Fig 7**). We obtained three distinct 3D class averages from the micrographs of the NANP_5.5_-1210-Fab-850 Fab complex, with angles of 110°, 130°, 155° between the Fabs (**Fig 7A**). In comparison, the analysis of 850 Fab-NANP_5_ micrographs yielded two distinct 3D class averages, with class 1 displaying the same angle (95°) between adjacent Fabs as in the 850-Fab-PfCSP cryoEM structure, and class 2 adopting an angle of 145° (**Fig 7B**). EM class averages of the 1210-NANP_5_ complex displayed wider angles of 130° and 155° [21] (**Fig 7C**). Therefore, it appears that complexes of 850 Fab-NANP_5_ and NANP_5.5_-1210-Fab-850 Fab can adopt similarly sharp angles between adjacent Fabs (95° and 110°, respectively), which are not observed in the 1210-NANP_5_ complex. We conclude that sharp angle interactions combined with extensive homotypic interactions between 850 Fab CDRs and FR residues allow for high-density packing around the PfCSP repeat, as observed in the high-density 850 Fab-PfCSP cryoEM structure.

## Discussion

The majority of anti-PfCSP repeat mAbs described to date were isolated after RTS,S/AS01 vaccination (e.g. mAb 311 and 317 [18], 397 [30], 239 and 399 [14]), after immunization with live Pf sporozoites under chloroquine prophylaxis (PfSPZ-CVac, e.g. mAb 1210 [17], 2541 and 4493 [12]), attenuated whole sporozoite vaccine (Sanaria PfSPZ, e.g. mAb CIS43 [27], MGG4 [13], L9 [31]), obtained from naturally infected patients (e.g. mAb 663 [32]) or after PfCSP immunization of humanized mice (e.g. mAbs 667 and 668 [33], iGL-CIS43.D3 [34]). mAb 850, identified in this study, is encoded by *IGHV3-33/IGKV1-5* germline genes, similarly to some of the most potent anti-malarial mAbs isolated to date, including 1210 [17], 239 [14], 2541 [12] and L9 [31]. Indeed, it was previously shown that the human humoral response following vaccination with RTS,S/AS01 [35] or PfSPZ-CVac [12,17,36,37] is dominated by *IGHV3-33/IGKV1-5*-encoded antibodies targeting NANP repeats. Many of these antibodies have limited SHM and contact the antigen mainly through germline-encoded residues, including *IGHV3-33* germline-encoded HC Trp52, a key HCDR2 residue for binding to NANP. mAb 850 utilizes H.Trp52 to interact with the NANP core motif and also has a characteristic 8-aa long KCDR3 [12,14,17]. mAb 850 possesses a SHM at position 31 of the HC that has also been observed in a number of *IGHV3-33* mAbs (311 [18], 1210 [17], 356 and 239 [14]), as well as the Val–Ile/Leu SHM at position 50 of the HC (mAbs 364 [14], 311 [18], MGG4 [13], 2541 and 4498 [12], S8 Fig). Presence of similar SHMs in antibodies isolated after different immunizations (including RTS,S/AS01, PfSPZ-CVac, and Sanaria PfSPZ), is evidence of a strong SHM selection at those positions, leading to improved affinity to PfCSP. These features observed for mAb 850 highlight the usefulness of the Kymouse platform in replicating human antibody responses to PfCSP by immunization.

In addition to antibodies against NANP repeats, some studies also identified antibodies that preferentially target the junction region that links the PfCSP N-terminus and the NANP repeats region, which contains a single NPDP motif and several interspersed NVDP motifs [13,27]. Cross-reactivity across repeat motifs is a feature of antibodies encoded by different Ig-gene combinations, and it was also observed for murine mAbs targeting CSP sequences from other species of *Plasmodium*, including Pb [20] and Pv [21]. Moreover, cross-reactivity appears to be associated with high binding affinity and parasite inhibition, and thus, repeated antigen exposure has been shown to result in the enrichment of cross-reactive antibodies [36]. Antibody selection in humans from sporozoite exposure is likely driven by affinity to NANP, rather than to NVDP or NPDP repeats [36], due to the higher number of NANP repeats in PfCSP [7] and differences in immunogenicity and accessibility of PfCSP epitopes [38,39]. Interestingly, mAb 850 was found to be partially cross-reactive to NVDP- and NPDP-containing peptides, despite being elicited by an immunogen that only contains NANP repeats. This observation supports the theory that cross-reactivity can be in part correlated with affinity to the NANP repeat for *IGHV3-33/IGKV1-5*-encoded antibodies [12]. Similar to other *IGHV3-33/IGKV1-5* mAbs [12], our structures suggest that mAb 850 cross-reactivity is achieved through the variable epitope aa’s pointing away from the paratope or being engaged by HCDR3 (**Fig 3C**). The ability of mAb 850 to bind N-junctional epitopes combined with its highly effective homotypic Fab-Fab contacts could be the main contributors to the high number of 850 Fabs binding to a single PfCSP molecule.

Indeed, biophysical and structural analyses revealed that mAb 850 binds PfCSP in a highly-dense immune complex that contains up to nineteen Fabs, with dense packing facilitated by extensive Fab-Fab homotypic contacts along the spiraling central repeat. To our knowledge, the 850 Fab-PfCSP complex is the highest molecular-weight PfCSP-Fab assembly reported to date, with other complexes containing up to fourteen Fabs, although the use of different recombinant PfCSP’s across studies only allows for relative comparisons [17,19,33,40]. Previous studies also suggest that regular, spiral assemblies can be induced by binding of either Fab or IgG to CSP, as shown for 311 mAb-PfCSP [19] and 3D11 mAb-PbCSP [20] complexes. Analysis of homotypic Fab interactions among *IGHV3-33*-encoded mAbs reveals different types of Fab-Fab contacts [12,14,17,19], with angles between adjacent Fabs ranging from 70 to 155° when bound to recombinant full-length PfCSP or corresponding peptides. In part, this diversity can be explained by the role of the heavy and light chain CDR’s in forming different contacts with adjacent Fabs. For example, the angle between adjacent 850 Fabs is sharper and Fab-Fab contacts encompass higher BSA compared to adjacent 1210 Fabs in its immune complex [17] (375 Å^2^ and 259 Å^2^, respectively). Interestingly, mAb 850 lacks somatic hypermutations at positions K.Lys93 and H.Lys56, which were shown to be crucial for Fab-Fab contacts in mAb 1210 [17]. Instead, mAb 850 utilizes largely germline residues, with addition of SHM residues and HCDR3 residues to form homotypic interactions (**Fig 6C**). Our biophysical experiments highlight the exquisitely high affinity of mAb 850 to the NANP repeat; they support the concept that germline-encoded residues at the sites of homotypic interactions are already amenable for high-affinity binding to the PfCSP NANP repeat and suggest that homotypic affinity maturation only played a minor role in driving the high affinity for the mutated antibody.

An important area of future investigation will be how homotypic interactions are selected at the B cell level during affinity maturation for enhanced BCR signaling [17] and how these can be leveraged favorably in immunization. In future studies, it will also be interesting to see how different immune-complex densities and modes of homotypic interactions impact PfCSP recognition anchored on the surface of sporozoites and how this might be linked to antibody inhibition of sporozoites. Due to the limited number of isolated mAbs, our study could not determine with confidence whether the NANP_5.5_-1210 Fab-LS-Th2R immunogen induced more antibodies that are capable of forming homotypic interactions compared to more conventional immunogens. Nonetheless, at polyclonal level, the NANP_5.5_-1210 Fab-LS-Th2R immunogen did not elicit an antibody response with higher inhibitory potency compared to more conventional immunogens where the NANP repeats remained untethered to a pre-immune complex. Thus, whether immunogen design approaches attempting to leverage homotypic interactions will improve the quality of the parasite inhibitory response against PfCSP still remains an unproven hypothesis to date.

mAb 850 is one of the highest-affinity NANP binders yet described, with a K_D_ of 1.0 X 10^−9^ M, 2.5 X 10^−11^ M and 3.2 X 10^−11^ M to the NPNA_3_ and NANP_6_ peptides, and recombinant full-length PfCSP, respectively. mAb 850 demonstrates potent inhibition of hepatocyte traversal by sporozoites with an IC_50_ of 1.1 μg/mL and *in vivo* efficacy in the liver burden model (~90% at 100 μg/mouse). Comparison of > 200 mAbs elicited by sporozoite immunization suggested that a NANP-affinity threshold at least in the nanomolar range was necessary for an antibody to be classified as inhibitory in *in vitro* traversal assays or in *in vivo* protection experiments in a mouse model of parasitemia [12]. A subsequent study suggested a direct correlation between apparent affinity against NANP repeats and *in vivo* efficacy in the liver burden assay [14]. mAb 850 satisfies these criteria for high-affinity binding and functionality, and therefore supports these models. Future studies continuing to explore large datasets of anti-PfCSP mAbs, also from different germline genes, will augment our understanding of the structural and biophysical properties of antibodies that associate best with *in vivo* protection against sporozoite infection.

## Materials and methods

### Ethics statement

All mice used in this study were housed and all procedures carried out under United Kingdom Home Office License 70/8718 with the approval of the Wellcome Trust Sanger Institute Animal Welfare and Ethical Review Body.

### Protein expression and purification

HC and KC variable regions of NANP_5.5_-1210 Fab, 850 Fab and H-57/99 850 mutant Fab were cloned (GeneArt) into custom pcDNA3.4 expression vectors immediately downstream of a human Igκ signal peptide and upstream of the Fab constant domain. pcDNA3.4-Fab KC and Fab HC plasmids were co-transfected into HEK293F cells for transient expression using FectoPRO (Polypus) or PEI MAX (Polysciences) DNA transfection reagents. Cells were cultured in GIBCO FreeStyle 293 Expression Medium for 5–7 days. NANP_5.5_-1210 Fab, 850 Fab and H-57/99 850 mutant Fab were purified via a combination of KappaSelect affinity chromatography (GE Healthcare), cation exchange chromatography (MonoS, GE Healthcare), and size-exclusion chromatography (Superdex 200 Increase 10/300 GL, GE Healthcare).

The NANP_5.5_-1210 Fab-LS-Th2R, NANP_5.5_-LS-Th2R and NANP_5.5_-ferritin-TSR constructs were cloned into the pHLsec plasmid with a Strep-tag. NANP_5.5_-1210 Fab-LS-Th2R pHLsec plasmid was transiently co-transfected with LS-pHLsec and 1210 Fab KC pcDNA3.4 plasmids at a 2:3:1 ratio in HEK293F cells using FectoPRO DNA transfection reagent (Polyplus). NANP_5.5_-LS-Th2R and NANP_5.5_-ferritin-TSR constructs were transiently co-transfected with either LS-pHLsec or ferritin-pHLsec at 1:1 ratio. Cells were cultured in GIBCO FreeStyle 293 Expression Medium for 6 days. Secreted nanoparticles were purified via StrepTrap (GE Healthcare) and size-exclusion chromatography (Superose 6 Increase 10/300 GL column, GE Healthcare).

Full-length PfCSP (strain NF54) was cloned into pcDNA3.4-TOPO with a His_6x_ tag. The resulting plasmid was transiently transfected in HEK293F cells using FectoPRO DNA transfection reagent (Polyplus). Cells were cultured in GIBCO FreeStyle 293 Expression Medium for 6 days. PfCSP was purified via HisTrap Ni-NTA (GE Healthcare) and size-exclusion chromatography (Superdex 200 Increase 10/300 GL column, GE Healthcare).

### CSP peptides

The synthetic peptides were custom made by CPC Scientific (NPNA_3_ (NPNANPNANPNA), NANP_6_ (NANPNANPNANPNANPNANPNANP), KQPADGNPDPNANPN, NPDPNANPNVDPNANP and NVDPNANPNVDPNANPNVDP)) and GenScript (NANP_3_ (NANPNANPNANP) and NANP_5_ (NANPNANPNANPNANPNANP)). With the exception of NANP_6_, all other peptides were acetylated at N-termini and amidated at C-termini. NANP_6_ contained an N-terminal biotin-aminohexanoic acid tag and an unmodified C-terminus.

### Cell lines

HEK293F cells (Thermo Fisher Scientific 12338026) were authenticated and validated to be mycoplasma-free by the commercial entity.

### Biolayer interferometry (BLI)

BLI (Octet RED96, ForteBio) experiments were performed to corroborate the binding of NANP_5.5_-1210 Fab-LS-Th2R nanoparticles to anti-CSP Fabs (4493 [12], 317 [18], CIS43 [27], 1210 [17], 2541 [12], 1710 [28]). All samples were diluted in kinetics buffer (PBS, pH 7.4, 0.01% [w/v] BSA, 0.002% [v/v] Tween-20) to 10 μg/mL and Fabs were immobilized onto anti-human Fab-CH1 biosensors (ForteBio). After a stable baseline was established, biosensors were dipped into wells containing NANP_5.5_-1210 Fab-LS-Th2R nanoparticles in kinetics buffer.

### Mouse immunization

A mix of male and female transgenic mice (mice from the Kymouse platform [22]) were immunised with NANP_5.5_-1210 Fab-LS-Th2R, NANP_5.5_-LS-Th2R, NANP_5.5_-ferritin-TSR nanoparticles or PfCSP in the presence of Sigma Adjuvant System (SAS, S6322, Sigma Aldrich). These mice from the Kymouse platform contain chimeric immunoglobulin loci, with humanized variable domains (V_H_, V_K_, and V_L_) and a humanized lambda constant domain (C_L_), but murine heavy (C_H_) and kappa (C_K_) constant domains. Kymab and Kymouse are trademarks of Sanofi Group. Mice were injected subcutaneously at base of tail at weeks 0, 3 and 6. Mice were sacrificed 7 days after the final boost under UK Home Office Schedule 1 (rising concentration of CO_2_). Spleen, lymph nodes, and bone marrow were collected and single cell suspensions cryopreserved. Whole blood was collected 1 week after each dose (weeks 1, 4 and week 7 terminal bleed). Serum was separated from hematocrit via centrifugation at 2000 g for 10 min, stored at −20°C and used to monitor titers by ELISA.

### Antibody isolation

Spleens, lymph nodes and bone marrow isolated from each mouse from the Kymouse platform were processed to single-cell suspensions, cryopreserved in 10% DMSO/FBS and stored in liquid nitrogen, prior to fluorescently-activated cell sorting (FACS) into individual wells of a 96 well plate in order to recover CD19+ B220+ GL7+ CD95+ germinal centre B cells from spleen and lymph nodes as well as CD138+ TACI+ plasma cells from spleen, lymph node and bone marrow samples (BD FACS Aria Fusion flow cytometer, Beckton Dickinson). Paired Ig genes were amplified by RT-PCR and Illumina libraries were generated before sequencing on an Illumina MiSeq. Heavy and light chain variable genes of selected antibodies were synthesized by Twist Biosciences (San Francisco, USA). Antibodies were recombinantly expressed as fully human IgG1 in HEK293 cells (Expi293F™ Cells, Gibco, Cat. No. A14635). Antibody supernatant was collected on day 8 after transfection and screened for binding by ELISA. Antibodies that bound CSP were re-expressed in suspension CHO-3E7 cells (NRC Canada) at a larger scale and purified using gravity flow columns (Econo-Pac Chromatography Columns, Bio-Rad, 732–1010) containing 1 mL MabSelect SuRe LX resin (GE Healthcare, 17-5474-03, P3303174) in order to generate enough material for further *in vitro* and *in vivo* assays.

### Enzyme-linked immunosorbent assay (ELISA)

Antigen ELISAs were performed as described [32]. In brief, high-binding 384-well polystyrene plates (Corning) were coated overnight at 4°C with 2 μg/mL of NANP_5_. Plates were washed three times with 0.05% Tween 20 in PBS, blocked with 50 μL of 4% BSA in PBS for 1 h at room temperature (RT), and washed again. For analysis of serum samples, sera were diluted in 1% BSA in PBS and 20 μL/well incubated for 90 min at RT. For analysis of monoclonal antibodies, 15 μL/well of serially diluted monoclonal antibodies (starting concentration 4.0 μg/mL, 1 in 4 dilution for 8 steps) were incubated for 2 h at RT. Wells were washed six times and incubated with goat anti-mouse IgG-HRP at 1:1000 (Jackson Immuno Research) in PBS with 1% BSA for 1 h. Wells were washed again and one-step ABTS substrate (RT, 20 μL/well; Roche) and 1× KPL ABTS peroxidase stop solution (RT, 20 μL/well; SeraCare Life Sciences) were used for detection. The concentration of antigen-specific IgG in serum was determined using an IgG1 standard curve (BD Pharming) on each plate. Area under the ELISA curve (AUC) was calculated using GraphPad Prism 7.04 (GraphPad).

### Surface plasmon resonance

The binding kinetics measurements of 317, 1210 and 850 mAbs interaction with PfCSP antigens were made using the Carterra LSA high-throughput surface plasmon resonance platform and CMD200M sensor chips (Carterra) at 25°C. Two microfluidic modules, a 96-channel print-head (96PH) and a single flow cell (SFC) were used to deliver liquids onto the sensor chip. In each assay, a single analyte antigen was titrated against the immobilized antibodies. The immobilization of antibodies onto the CMD200M chips depended on the type of analyte used during titration. In assays involving full-length PfCSP used as an analyte, a goat anti-Human IgG Fc antibody (Millipore) was first immobilized onto the chip through amine-coupling. The chip was first activated by 100 mM N-Hydroxysuccinimide (NHS) and 400 mM 1-Ethyl-3-(3-dimethylaminopropyl) carbodiimide hydrochloride (EDC) (GE healthcare, mixed 1:1:1 with 0.1 M MES buffer at pH 5.5) for 600 s, followed by immobilization of anti-Human IgG Fc (in 10 mM Sodium Acetate at pH 4.5) at 50 μg/ml for 900 s. Unreactive esters were quenched with 600 s injection of 1 M Ethanolamine-HCl at pH 8.5. The chip was then exposed to double pulses (30 s per pulse) of 10 mM Glycine at pH 2.0. The PfCSP-specific mAbs were then captured on anti-Hu IgG Fc surfaces by injection of mAbs at 10 μg/ml or 5 μg/ml concentration for 600 s using the 96PH, with 1X HBSTE buffer (10 mM HEPES pH 7.4, 150 mM NaCl, 3 mM EDTA and 0.01% Tween-20) as running buffer and antibody diluent. If PfCSP peptide antigens were used as analytes, the chip was activated by NHS/EDC for 600 s, followed by direct immobilization of PfCSP-specific mAbs (in 10 mM Sodium Acetate at pH 4.5) injected at 10 μg/ml or 5 μg/ml concentrations for 600 s using the 96PH. Unreactive esters were then quenched with a 600 s injection of 1 M ethanolamine-HCl at pH 8.5. Then 45 cycles of 1X HBSTE buffer injections with 1X HBSTE also as running buffer were used to wash off non-specifically bound IgG overnight from the sensor chip surface without using regeneration buffer. Except for the capture of mAbs by anti-Human IgG Fc and washing of non-specifically bound IgG, the running buffer was 10 mM MES buffer at pH 5.5 with 0.01% Tween-20, and each PfCSP-specific mAb at a given diluted concentration was immobilized onto 4 separate spots of the same chip, enabling replicating binding kinetics measurements. Unless specified above, the steps were done using the SFC.

A two-fold dilution series of the antigen was prepared in 1x HBSTE buffer. The top concentration for full-length PfCSP and all PfCSP peptide antigens was 8 μg/ml (0.25 μM for CSP, 2.92 μM for NANP_6_, 6.41 μM for NPNA_3_, 3.76 μM for NVDPNANPNVDPNANPNVDP, 4.70 μM for NPDPNANPNVDPNANP, 5.03 μM for KQPADGNPDPNANPN). The antigen at different concentrations was then injected using SFC onto the chip surface from the lowest to the highest concentration without regeneration, including 8 injections of buffer before the lowest non-zero concentration for signal stabilization. For each concentration, the data collection involved 120 s of baseline step and 900 s of dissociation steps. The duration of association step was 240 s for full-length PfCSP and NANP_6_, and was 300 s for all other PfCSP peptide antigens. For all assays the running buffer for titration was 1X HBSTE.

The kinetics titration data collected were first pre-processed in the NextGenKIT (Carterra) software, including reference subtraction, buffer subtraction and data smoothing. The data were then exported and analyzed using the TitrationAnalysis tool developed in-house [41]. The specific binding time courses for each antibody construct immobilized on different spots were fitted to a 1:1 Langmuir model to derive *k*_*a*_, *k*_*d*_ and *K*_*D*_ values. For antigens with multiple repeats of epitopes, the *K*_*D*_ values determined includes avidity effect. For each antibody–antigen pair, the best triplicate measurements satisfying the pre-set data acceptance criteria were selected. The pre-set acceptance criteria for quality control included 1) standard error of the estimated *k*_*a*_, *k*_*d*_ and *K*_*D*_ in each replicate ≤20% and 2) fold change for *k*_*a*_, *k*_*d*_ and *K*_*D*_ values within the triplicate ≤ 3.

### Traversal assay

*Anopheles coluzzii* (Ngousso strain) mosquitoes were kept at 28°C 70–80% humidity and 12/12–h day/night cycle. The Pf NF54 clone used in this study originated from Prof. Sauerwein’s laboratory (Radboud University Medical Center, Nijmegen, Netherlands) and was tested for mycoplasma contamination regularly. For infections, mosquitoes were fed for 15 min on a membrane feeder containing Pf mature gametocytes cultured with O+ human red blood cells (Haema, Berlin) and thereafter kept in a secured BSL3 laboratory according to national regulations (Landesamt für Gesundheit und Soziales; project no. 297/13). Unfed mosquitoes were removed shortly after infections. Blood-fed mosquitoes were maintained at 26°C 70–80% humidity and offered an additional uninfected blood meal 8 days after infection. Salivary gland sporozoites were isolated from mosquito thoraxes 13–15 days after infection in HC-04 complete medium (MEM without L-glutamine (Gibco) supplemented with F-12 Nutrient Mix with L-glutamine (Gibco) in a 1:1 ratio, 15 mM HEPES, 1.5 g/L NaHCO_3_, 2.5 mM additional L-glutamine, and 10% FCS) and kept on ice until further use. Traversal assays were performed as previously described [36]. Briefly, mAbs at the indicated concentrations or pooled serum samples from immunized mice (1:100 dilution) were incubated with 50,000 salivary gland sporozoites in HC04 medium for 30 min on ice. After incubation, dextran-rhodamine (0.5 mg/mL, Molecular Probes) was added at 0.5 mg/mL before being transferred onto the human hepatocyte cell line HC04 [42], and sporozoites were incubated with the cells for 2 h at 37°C. After washing, trypsinization and fixation with 1% paraformaldehyde, dextran positivity of the cells was measured by FACS LSR II instrument (BD Biosciences). Background detected in cells treated with uninfected mosquito salivary gland material was subtracted and the data was normalized to maximum traversal capacity in the absence of antibodies. Data were analyzed using FlowJo V.10.0.8 (Tree star). Humanized version of murine anti-NANP mAb 2A10 [32], and non-Pf reactive mAb mGO53 [43], were used as positive and negative controls, respectively.

### Liver burden

Parasite challenge to measure liver burden was assessed as described [29]. Briefly, 6-8-week-old C57Bl/6 female mice were injected intravenously with 100 μg of the respective mAb. Mice were challenged 16 h later with 2,000 PfCSP-expressing transgenic Pb sporozoites delivered i.v. in HBSS-2% FBS. Mice were injected intraperitoneally with 100 μl of D-luciferin at 30 mg/mL 42 h after sporozoite challenge, anesthetized with isoflurane and imaged in the IVIS Spectrum Imaging System to measure the bioluminescence expressed by the transgenic parasite.

### Isothermal titration calorimetry (ITC)

ITC experiments were performed with an Auto-iTC200 instrument (Malvern) at 25°C. Titrations were performed with WT 850 or H-57/99 mutant Fab in the syringe in 15 successive injections of 2.5 μL or 20 successive injections of 2.0 μL. Full-length recombinant PfCSP, NANP_3_ or NANP_5_ was added to the calorimetric cell. 850 WT Fab, 850 H-57/99 Fab, PfCSP, NANP_3_ and NANP_5_ were diluted in Tris-buffered saline (TBS; 20 mM Tris pH 8.0, and 150 mM NaCl). PfCSP was diluted to 13 μM and titrated with Fab at 1340 μM. NANP_3_ was diluted to 5–10 μM and titrated with Fab at 75–100 μM. NANP_5_ was diluted to 7.5–10 μM and titrated with Fab at 150–200 μM. Experiments were performed in at least duplicate, and the mean and standard error of the mean are reported. ITC data were analyzed using the Micro-Cal ITC Origin 7.0 Analysis Software according to a 1:1 binding model.

### Size-exclusion chromatography-multi-angle light scattering (SEC-MALS)

Full-length recombinant PfCSP was complexed with a molar excess of 850 Fab and loaded on a Superose 6 Increase 10/300 GL column (GE Healthcare) using an Agilent Technologies 1260 Infinity II HPLC coupled in-line with the following calibrated detectors: (i) MiniDawn Treos MALS detector (Wyatt); (ii) Quasi elastic light scattering (QELS) detector (Wyatt); and (iii) Optilab TreX refractive index (RI) detector (Wyatt). Data processing was performed using the ASTRA software (Wyatt).

### Crystallization and structure determination

850 Fab was incubated with a V_H_H nanobody [44] at 1:2 molar ratio and purified via size-exclusion chromatography (Superdex 200 Increase 10/300 GL, GE Healthcare). The 850 Fab-V_H_H complex was concentrated to 10 mg/mL and entered into crystallization trials in sitting drop vapor diffusion experiments either directly, or after mixing with NANP_3_ peptide in a 1:3 molar ratio. X-ray data were collected from crystals obtained in 85 mM Tris pH 8.5, 170 mM sodium acetate, 25.5% (w/v) polyethylene glycol 4000, 15% glycerol for the 850 Fab-V_H_H complex, and in 0.2 M KCl, 20% (w/v) polyethylene glycol 2250 for the 850 Fab-V_H_H-NANP_3_ complex. 850 Fab-V_H_H-NANP_3_ crystals were cryoprotected in 25% (v/v) ethylene glycol before being flash-frozen in liquid nitrogen, while 850 Fab-V_H_H crystals were flash-frozen without additional cryoprotectant.

Data were collected at the 23-ID-D beamline at the Argonne National Laboratory Advanced Photon Source. All datasets were processed and scaled using XDS [45]. Structures were determined by molecular replacement using Phaser [46]. Refinement of structures was performed using phenix.refine [47] and iterations of refinement using Coot [48]. Access to all software was supported through SBGrid [49]. Interactions were analyzed using the PDBePisa server [50].

### CryoEM data collection and image processing

The 850 Fab-PfCSP complex was concentrated to 0.5 mg/mL and 3.0 μl of the sample was applied on homemade holey gold grids [51], which were glow-discharged in air for 15 s before use. The sample was blotted for 12.5 s, and subsequently plunge-frozen in a mixture of liquid ethane and propane [52] using a modified FEI Vitrobot (maintained at 4°C and 100% humidity). Data collection was performed on a Thermo Fisher Scientific Titan Krios G3 operated at 300 kV with a prototype Falcon 4 camera automated with the EPU software. A nominal magnification of 75,000× (calibrated pixel size of 1.03 Å) and defocus range between 0.8 and 2.5 μm were used for data collection. Exposures were collected for 9.6 s as movies of 30 frames with a camera exposure rate of ∼5 e^−^ per pixel per second, and total exposure of ~45 electrons/Å^2^. A total of 4306 raw movies were obtained.

Image processing was carried out in cryoSPARC v2 [53]. Initial specimen movement correction, exposure weighting, and CTF parameters estimation were done using patch-based algorithms. Manual particle selection was performed on 30 micrographs to create templates for template-based picking. 1,295,064 particle images were selected by template picking and individual particle images were corrected for beam-induced motion with the local motion algorithm [54]. *Ab-initio* structure determination revealed that most particles images present in the dataset correspond to the 850 Fab-PfCSP complex, with a minor population corresponding to unbound 850 Fab. After several rounds of heterogeneous refinement, 165,747 particle images were selected for non-uniform refinement [55] with no symmetry applied, which resulted in a 3.4 Å resolution map of the 850 Fab-PfCSP complex estimated from the gold-standard refinement with correction of the Fourier shell correlation (FSC) for masking effects.

An initial model for 850 Fab-PfCSP was created by manually docking fourteen copies of the 850 Fab crystal structure into the 850 Fab-PfCSP cryoEM map using UCSF Chimera [56], followed by manual building using Coot [48]. All models were refined using phenix.real_space_refine [57] with secondary structure and geometry restraints. The final models were evaluated by MolProbity [58]. The figures were prepared with UCSF Chimera [56] and UCSF ChimeraX [59]. Interactions in the 850 Fab-PfCSP complex were identified by PDBePISA [50].

### Negative-stain EM

850 Fab-NANP_5_ and NANP_5.5_-1210 Fab-850 Fab complexes were purified on a Superdex 200 Increase 10/300 GL column (GE Healthcare) and diluted to 10 μg/mL. NANP_5.5_-1210 Fab-LS-Th2R nanoparticles were purified on a Superose 6 Increase 10/300 GL column (GE Healthcare) and diluted to 50 μg/mL. All samples were applied onto homemade carbon film-coated grids and stained with 2% uranyl formate. Specimens were imaged with a FEI Tecnai T20 electron microscope operating at 200 kV with an Orius charge-coupled device (CCD) camera (Gatan Inc). A calibrated 34,483 x magnification, resulting in a pixel size of 2.71 Å, was used for data collection. Particle selection, extraction and three rounds of 2D classification with 50 classes were performed with Relion [60] and cryoSPARC v2 [53].

## Supporting information

S1 Fig NANP_5.5_-1210 Fab-LS-Th2R nanoparticles and comparator immunogens (NANP_5.5_-LS-Th2R, NANP_5.5_-ferritin-TSR, and PfCSP) induce functional antibodies in mice from the Kymouse platform. (**A**) Anti-NANP IgG titer measured by ELISA. Each dot represents an individual mouse. (**B**) Inhibition of Pf sporozoite traversal activity by the post-immune sera (1:100 dilution, 7 days post final boost, pooled serum samples by immunogen group). Each dot represents one experimental replicate and black horizontal lines indicate arithmetic means.
(TIF)

S2 Fig PfCSP affinity and gene usage of mAbs isolated after immunization with NANP_5.5_-1210 Fab-LS-Th2R nanoparticles. (**A**) PfCSP ELISA reactivity of monoclonal germinal center B cell- and plasma cell-derived antibodies (n = 113). Data in A are representative at least two independent experiments. (**B**) Frequency of Ig gene combination observed in PfCSP-reactive (top) and non-reactive (bottom) mAbs.
(TIF)

S3 Fig Binding characteristics and *in vitro* sporozoite traversal inhibition of isolated mAbs. (**A**) Affinity profiles of mAbs to NPNA_3_ peptide measured by SPR. Black lines indicate geometric mean. Pf traversal assays were performed at 1.0 μg/mL (**B**) and 0.5 μg/mL (**C**). Each dot represents one experimental replicate and the black horizontal lines in **B**–**C** indicate arithmetic means. Humanized versions of murine mAbs 2A10 [32] and mGO53 [43] were used at 100 μg/mL as technical positive and negative controls, respectively.
(TIF)

S4 Fig Representative sensorgrams and kinetics summaries of 317, 1210 and 850 binding to full-length PfCSP and PfCSP peptide antigens. The mAb label associated with each sensorgram is indicated at the top of the column where the sensorgram is located; the antigen label associated with each sensorgram is indicated to the left of the row where the sensorgram is located. Each sensorgram is a representative sensorgram chosen from the triplicate measurements for the same antibody-antigen pair. For titrations showing strong binding, each representative sensorgram is overlaid with its best fit, with the averages and the standard deviations of *k*_*a*_, *k*_*d*_ and K_*D*_ for the triplicate measurements shown underneath the respective sensorgram. For titrations showing weak or no binding, representative sensorgrams without fits are shown. The numbers to the right of each sensorgram show the nanomolar concentrations of analyte used during titration cycles. The shared axes labels are shown at the bottom and on the left side of the sensorgram grid.
(TIF)

S5 Fig Binding cross-reactivity of mAb 850 in comparison to mAbs 1210 and 317 as measured by SPR. Red dashed line represents binding affinity of 10 μM. Binding affinities weaker than 10 μM and no binding are represented by symbols on the red dashed line. P values were calculated by Mann–Whitney test. *P < 0.05.
(TIF)

S6 Fig Stereo-image of the composite omit map electron density obtained from crystal structures. Map contoured at 1.0–1.2 sigma for the HCDR3 in 850 Fab crystal structure (**A**), HCDR3 in 850 Fab-NANP_3_ crystal structure (**B**), NANP_3_ peptide in 850 Fab-NANP_3_ crystal structure (**C**).
(TIF)

S7 Fig Comparison between the 850 Fab, 850 Fab-NANP_3_ and 1210 Fab-NANP_5_ crystal structures. (**A**) Color representation of the all-atom RMSD between the 850 Fab and 850 Fab-NANP_3_ crystal structures. (**B**) Differences in HCDR3 conformation between the 850 Fab and 850 Fab-NANP_3_ crystal structures. (**C**) Color representation of the backbone RMSD between 850 Fab-NANP_3_ and 1210 Fab-NANP_5_ crystal structures. Models were aligned using PyMOL [61]. RMSD values were calculated using UCSF Chimera [56] and plotted by color on the secondary structure of the 850 Fab (**A**) or 850 Fab-NANP_3_ crystal structure (**C**).
(TIF)

S8 Fig Sequence alignment of selected PfCSP-reactive mAbs. HCs of mAb 850 and other *IGHV3-33*-encoded mAbs (**A**, 311 [18], 356, 239 [14], MGG4 [13], 2243, 2541, 4498, 3945 [12], and 1210 [17]) and KCs of mAb 850 and other *IGHV3-33/IGKV1-5*-encoded mAbs (**B**, 239 [14], 2541, 2243 [12] and 1210 [17]) with their germline *VH* and *VK* Ig gene segments. (*) denotes mAbs encoded by *IGHV3-33* and *IGKV1-5*-genes, (.) denotes mAb residues identical to the germline precursor, and yellow highlight denotes residues involved in PfCSP recognition. Values in brackets indicate percent identity between mAbs and their germline genes.
(TIF)

S9 Fig CryoEM data processing workflow in cryoSPARC v2. (TIF)

S10 Fig CryoEM analysis of the 850 Fab-PfCSP complex. (**A**) Left panel–a representative cryoEM micrograph of the 850 Fab-PfCSP complex from a 200 kV screening microscope with individual particles highlighted with white circles. Scale bar: 50 nm. (**B**) Selected 2D class averages of the 850 Fab-PfCSP complex. (**C**) Particle orientation distribution plot. (**D**) Fourier shell correlation curve from the final 3D non-uniform refinement of the 850 Fab-PfCSP complex in cryoSPARC v2. (**E**) Local resolution (Å) plotted on the surface of the cryoEM map. (**F**) CryoEM map of PfCSP (grey mesh) with the model shown as sticks (black carbons).
(TIF)

S11 Fig Color representation of the backbone RMSD of the Fab variable region and NANP repeats between the 850 Fab-PfCSP cryoEM structure and the 850 Fab-NANP_3_ crystal structure. Models were aligned using PyMOL [61]. RMSD values were calculated using UCSF Chimera [57] and plotted by color on the secondary structure of the 850 Fab-PfCSP cryoEM structure.
(TIF)

S12 Fig Homotypic contacts between adjacent 850 Fabs in 850 Fab-PfCSP cryoEM structure. (**A**) Interface between Fabs A and C includes FR1 and FR3 of Fab A HC and FR1 and FR3 of Fab C KC, as well as two residues in CDR regions (K.Ser52 from KCDR2 and H.Glu99 from HCDR3 of Fab C). H-bonds are represented by dash lines. (**B**) Amino acid sequence alignment of mAbs 850 with the germline *VH* (top) and *VK* (bottom) Ig gene segments. Yellow highlight: germline-encoded residues involved in homotypic interactions; green highlight: somatically hypermutated residues involved in homotypic interactions that do not form interactions with PfCSP. (**C**) Interface between Fabs A and D. Black dashed line denotes H-bond. Residues forming Fab-Fab contacts are labeled with the position of the Fab (A and D) indicated in subscript. (**D**) Amino acid sequence alignment of mAbs 850 with the germline *VH* (top) and *VK* (bottom) Ig gene segments. Yellow highlight: germline-encoded residues involved in homotypic interactions.
(TIF)

S13 Fig Binding of 850 Fab and H-57/99 germline-reverted mutant Fab to NANP peptides. ITC measurements of 850 Fab and H-57/99 Fab binding to NANP_3_ (**A** and **B**, respectively), and 850 Fab and H-57/99 Fab binding to NANP_5_ (**C** and **D,** respectively) at 25°C. Representative raw data are shown above the corresponding plot and trendline of heat of injectant. Mean K_D_ values resulting from at least duplicate experiments are indicated with the associated standard error of the mean (SEM). Stoichiometry (N) values were consistently found to be greater for NANP_5_ than NANP_3_ but are not reported due to large variability associated with measurement of peptide concentrations.
(TIF)

S1 Video 3D variability analysis of 850 Fab-PfCSP complex. (MP4)

S1 Table PfCSP-reactive antibody gene features. (DOCX)

S2 Table Summary of peptides used in this study. (DOCX)

S3 Table Table of contacts between 850 Fab and NANP_3_ peptide. (DOCX)

S4 Table Table of contacts between one of the 850 Fabs and PfCSP in the 850 Fab-PfCSP complex. (DOCX)

S5 Table Homotypic Fab-Fab contacts in the 850 Fab-PfCSP complex. (DOCX)
