# Supplementary figures and images for: High-density binding to Plasmodium falciparum circumsporozoite protein repeats by inhibitory antibody elicited in mouse with human immunoglobulin repertoire
Source: PLoS Pathog. 2022 Nov 28;18(11):e1010999. doi: 10.1371/journal.ppat.1010999 (PMC9762590; doi:10.1371/journal.ppat.1010999)

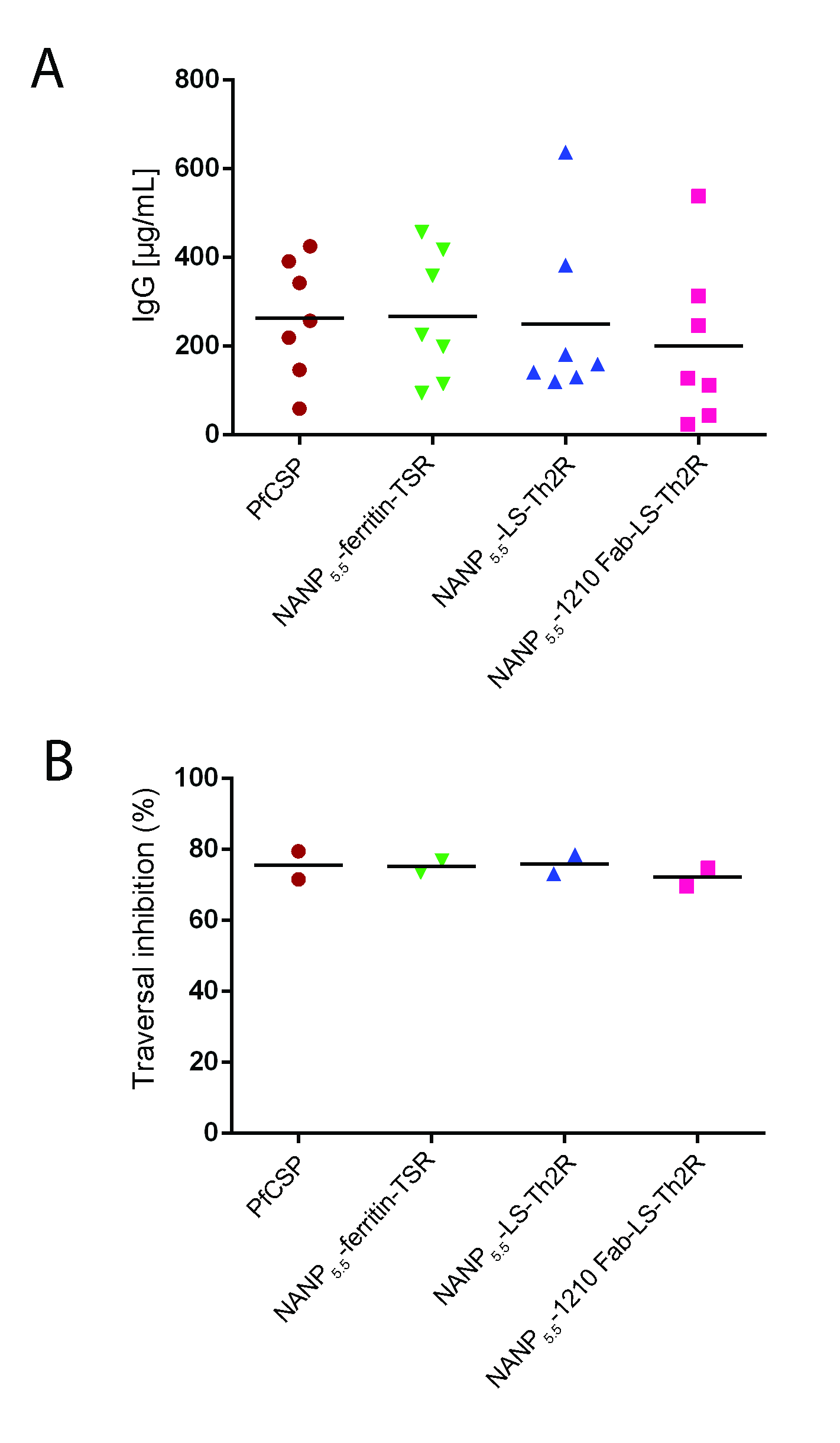

Supplement: S1 Fig — (A) Anti-NANP IgG titer measured by ELISA. Each dot represents an individual mouse. (B) Inhibition of Pf sporozoite traversal activity by the post-immune sera (1:100 dilution, 7 days post final boost, pooled serum samples by immunogen group). Each dot represents one experimental replicate and black horizontal lines indicate arithmetic means. (TIF) [file ppat.1010999.s001.tif]

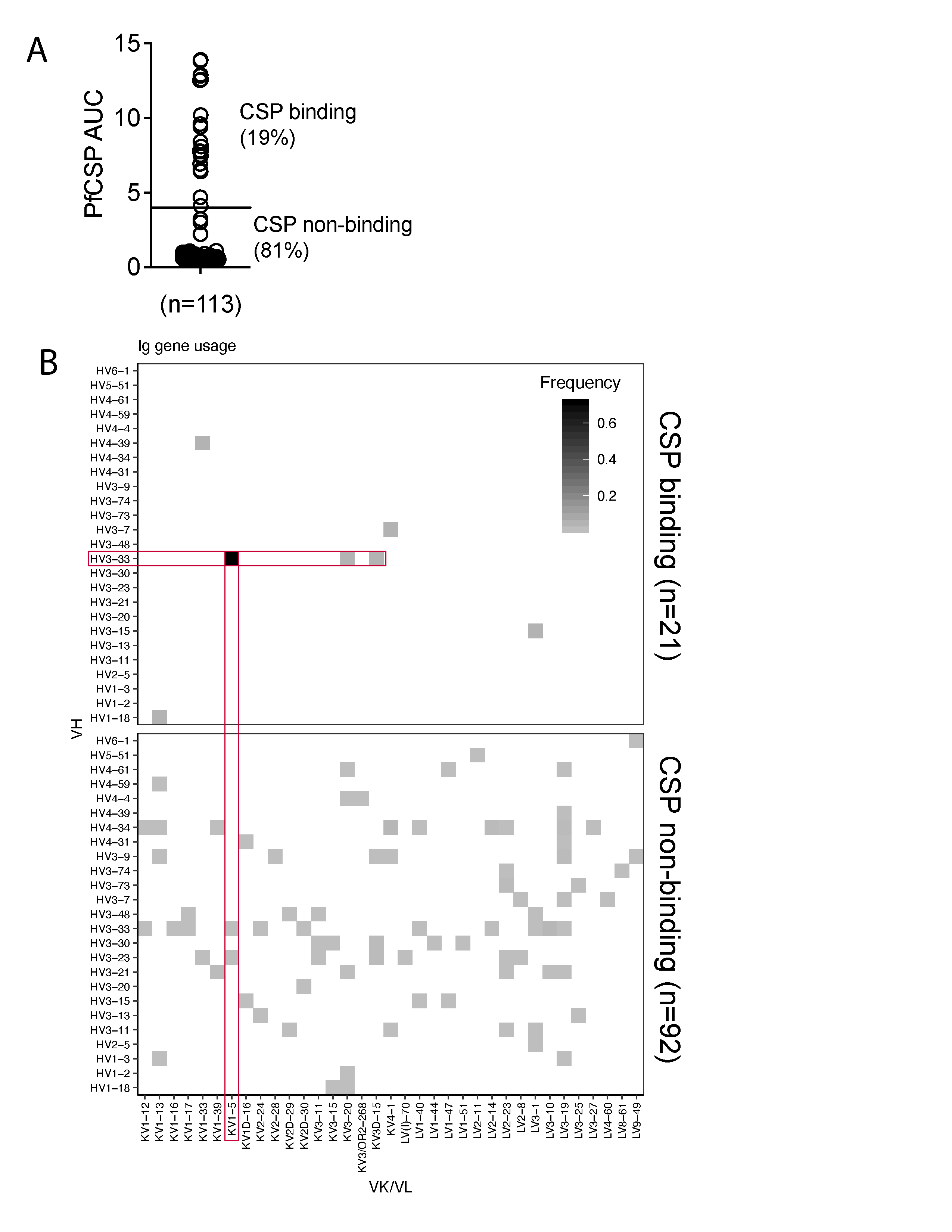

Supplement: S2 Fig — (A) PfCSP ELISA reactivity of monoclonal germinal center B cell- and plasma cell-derived antibodies (n = 113). Data in A are representative at least two independent experiments. (B) Frequency of Ig gene combination observed in PfCSP-reactive (top) and non-reactive (bottom) mAbs. (TIF) [file ppat.1010999.s002.tif]

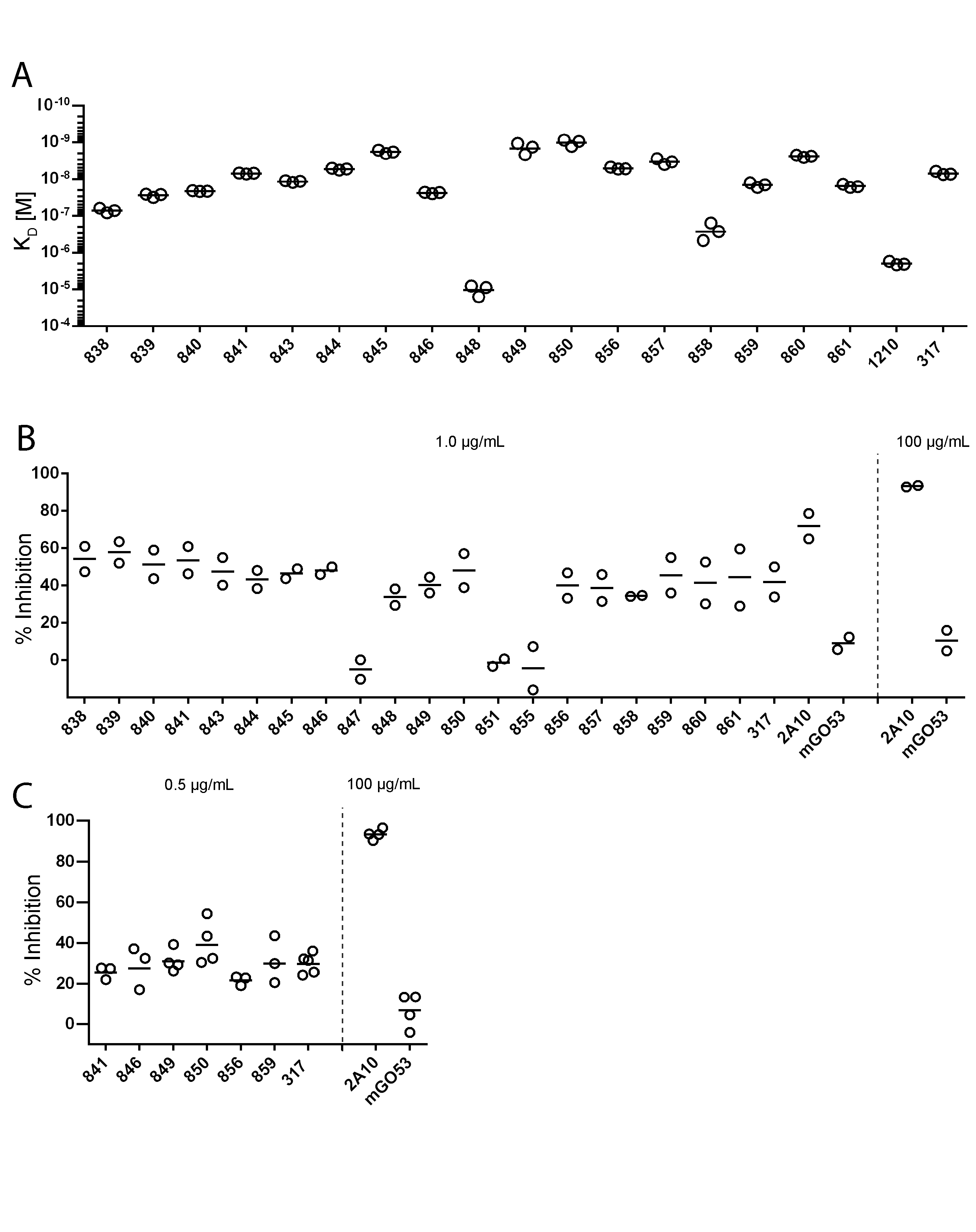

Supplement: S3 Fig — (A) Affinity profiles of mAbs to NPNA3 peptide measured by SPR. Black lines indicate geometric mean. Pf traversal assays were performed at 1.0 μg/mL (B) and 0.5 μg/mL (C). Each dot represents one experimental replicate and the black horizontal lines in B–C indicate arithmetic means. Humanized versions of murine mAbs 2A10 [32] and mGO53 [43] were used at 100 μg/mL as technical positive and negative controls, respectively. (TIF) [file ppat.1010999.s003.tif]

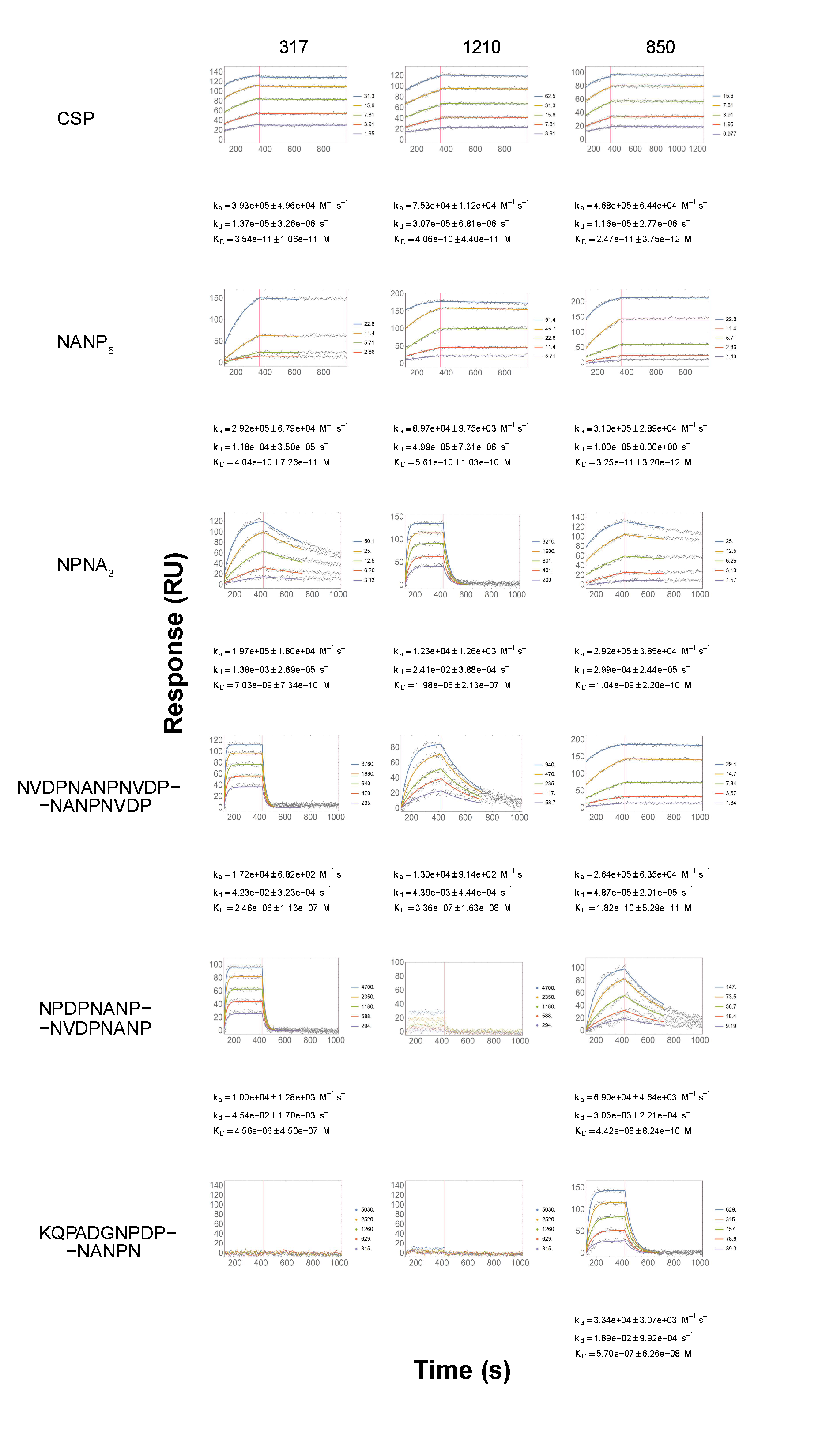

Supplement: S4 Fig — The mAb label associated with each sensorgram is indicated at the top of the column where the sensorgram is located; the antigen label associated with each sensorgram is indicated to the left of the row where the sensorgram is located. Each sensorgram is a representative sensorgram chosen from the triplicate measurements for the same antibody-antigen pair. For titrations showing strong binding, each representative sensorgram is overlaid with its best fit, with the averages and the standard deviations of ka, kd and KD for the triplicate measurements shown underneath the respective sensorgram. For titrations showing weak or no binding, representative sensorgrams without fits are shown. The numbers to the right of each sensorgram show the nanomolar concentrations of analyte used during titration cycles. The shared axes labels are shown at the bottom and on the left side of the sensorgram grid. (TIF) [file ppat.1010999.s004.tif]

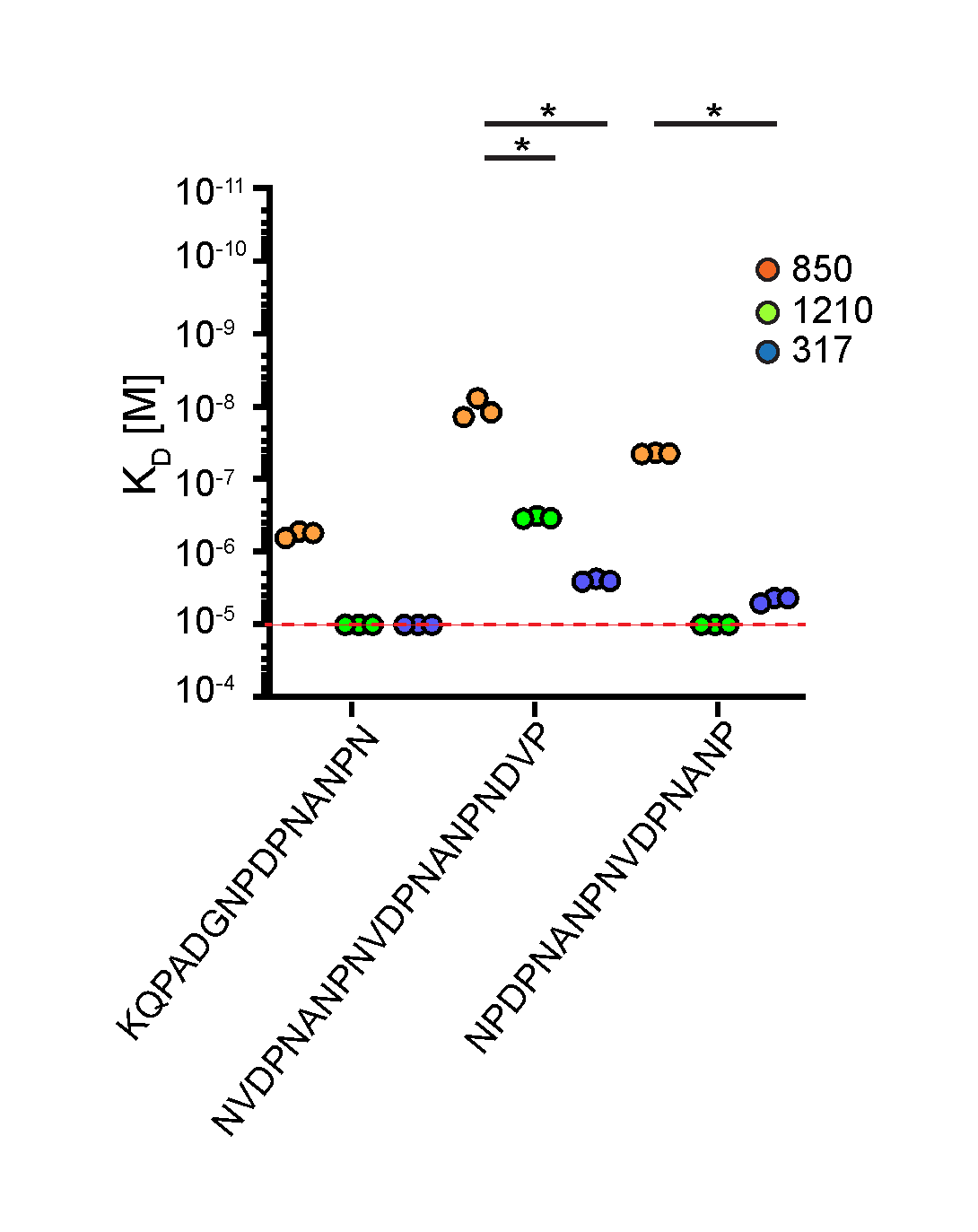

Supplement: S5 Fig — Red dashed line represents binding affinity of 10 μM. Binding affinities weaker than 10 μM and no binding are represented by symbols on the red dashed line. P values were calculated by Mann–Whitney test. *P < 0.05. (TIF) [file ppat.1010999.s005.tif]

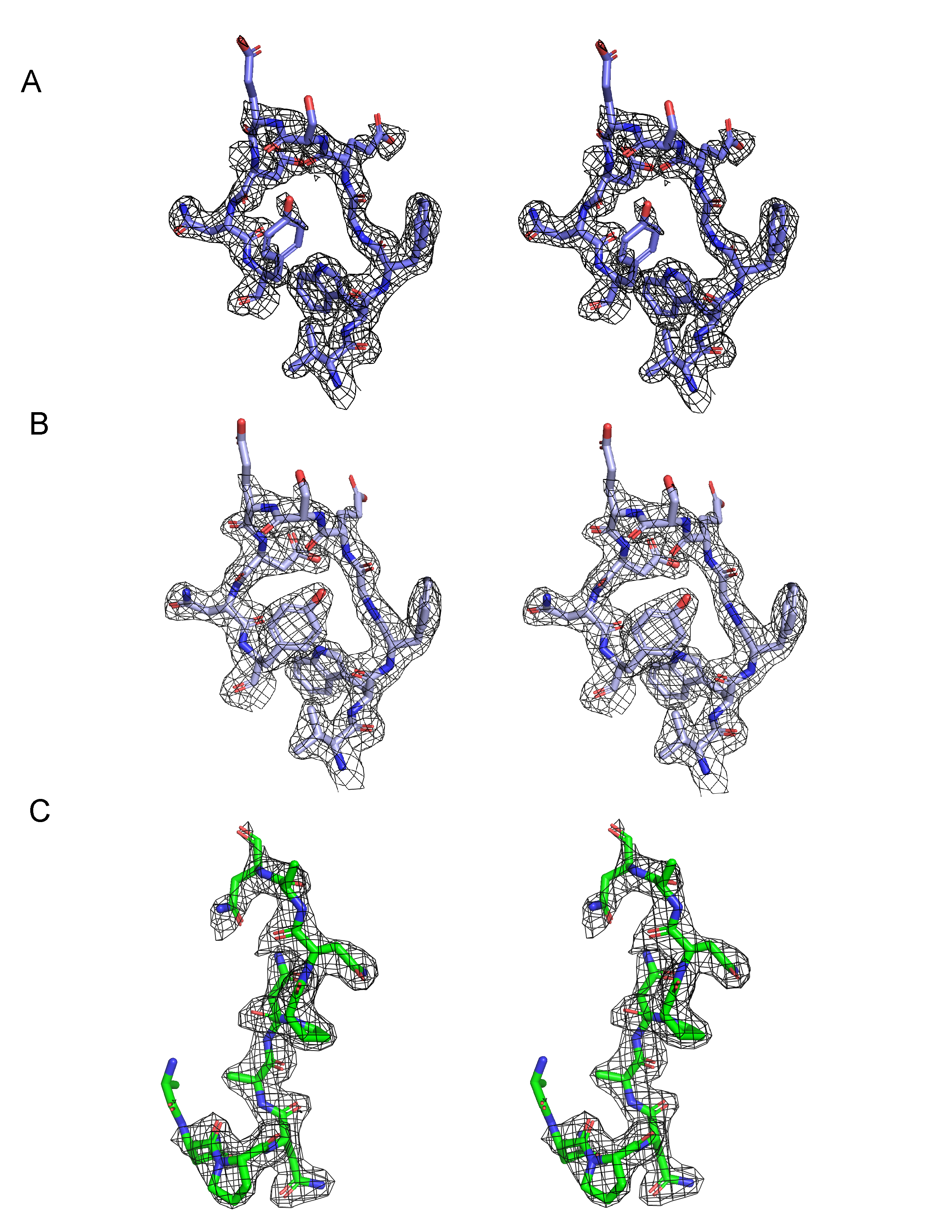

Supplement: S6 Fig — Map contoured at 1.0–1.2 sigma for the HCDR3 in 850 Fab crystal structure (A), HCDR3 in 850 Fab-NANP3 crystal structure (B), NANP3 peptide in 850 Fab-NANP3 crystal structure (C). (TIF) [file ppat.1010999.s006.tif]

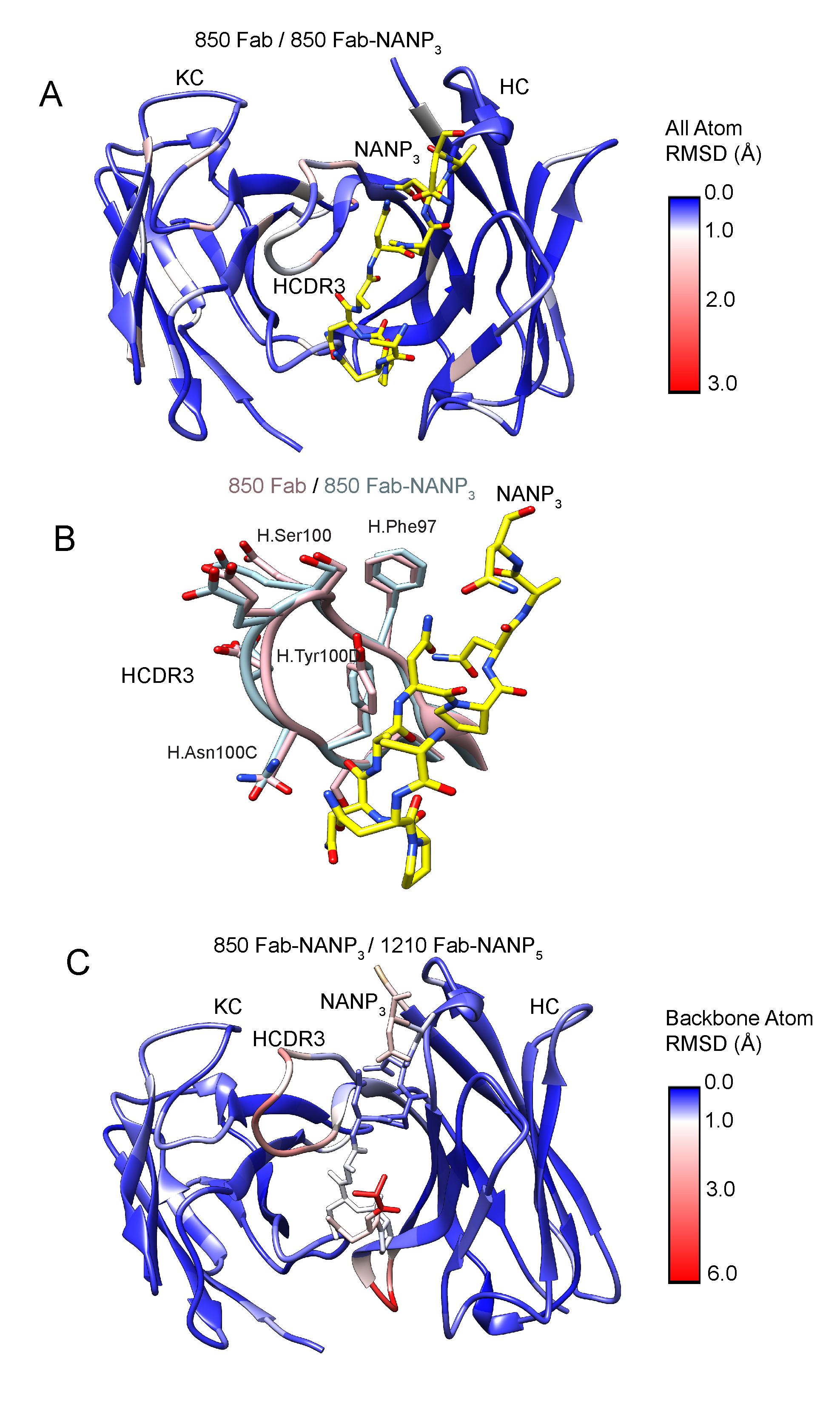

Supplement: S7 Fig — (A) Color representation of the all-atom RMSD between the 850 Fab and 850 Fab-NANP3 crystal structures. (B) Differences in HCDR3 conformation between the 850 Fab and 850 Fab-NANP3 crystal structures. (C) Color representation of the backbone RMSD between 850 Fab-NANP3 and 1210 Fab-NANP5 crystal structures. Models were aligned using PyMOL [61]. RMSD values were calculated using UCSF Chimera [56] and plotted by color on the secondary structure of the 850 Fab (A) or 850 Fab-NANP3 crystal structure (C). (TIF) [file ppat.1010999.s007.tif]

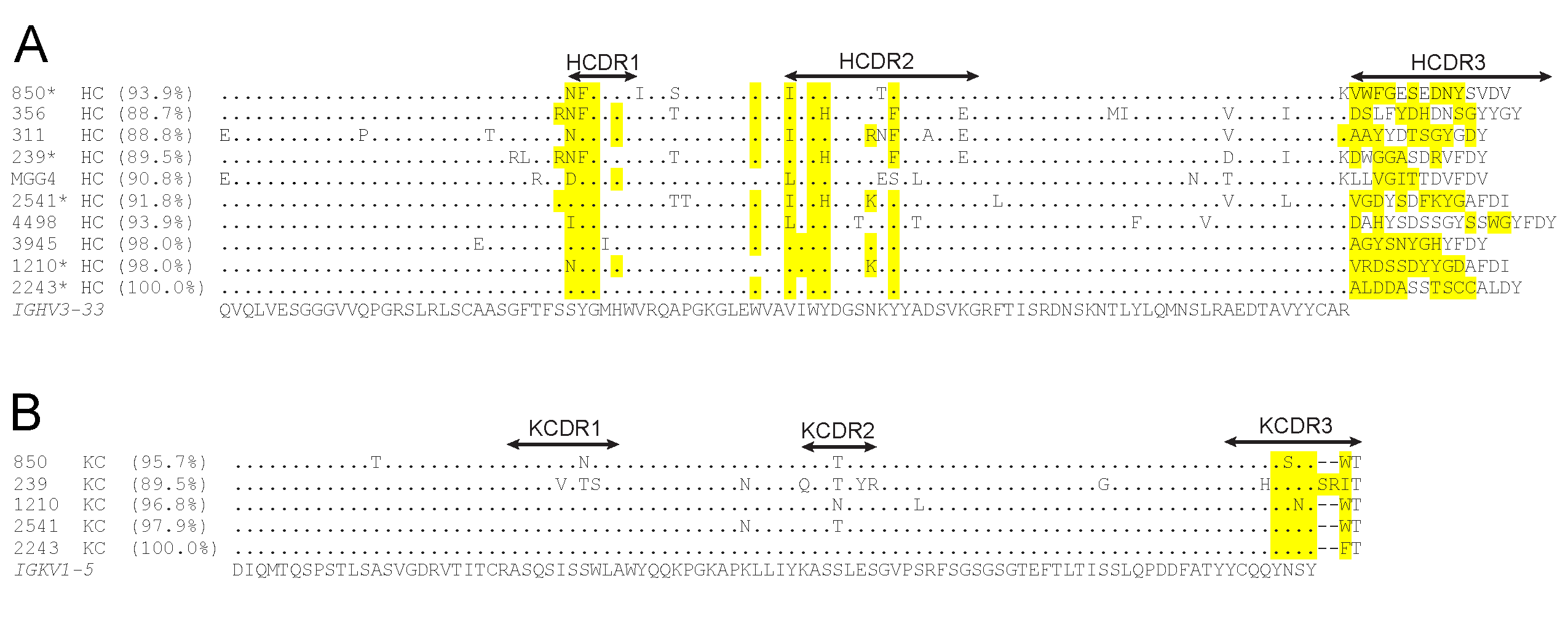

Supplement: S8 Fig — HCs of mAb 850 and other IGHV3-33-encoded mAbs (A, 311 [18], 356, 239 [14], MGG4 [13], 2243, 2541, 4498, 3945 [12], and 1210 [17]) and KCs of mAb 850 and other IGHV3-33/IGKV1-5-encoded mAbs (B, 239 [14], 2541, 2243 [12] and 1210 [17]) with their germline VH and VK Ig gene segments. (*) denotes mAbs encoded by IGHV3-33 and IGKV1-5-genes, (.) denotes mAb residues identical to the germline precursor, and yellow highlight denotes residues involved in PfCSP recognition. Values in brackets indicate percent identity between mAbs and their germline genes. (TIF) [file ppat.1010999.s008.tif]

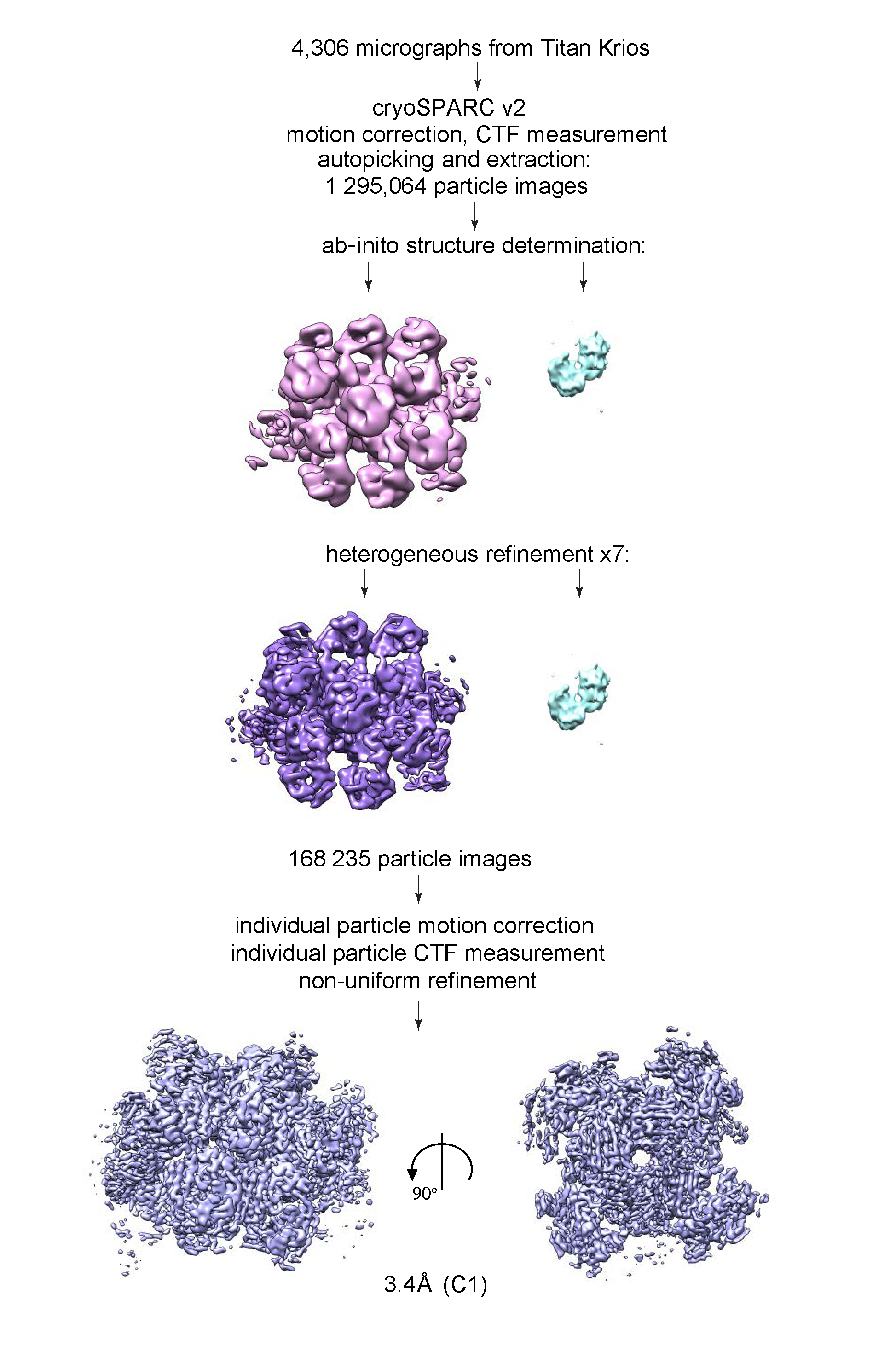

Supplement: S9 Fig — (TIF) [file ppat.1010999.s009.tif]

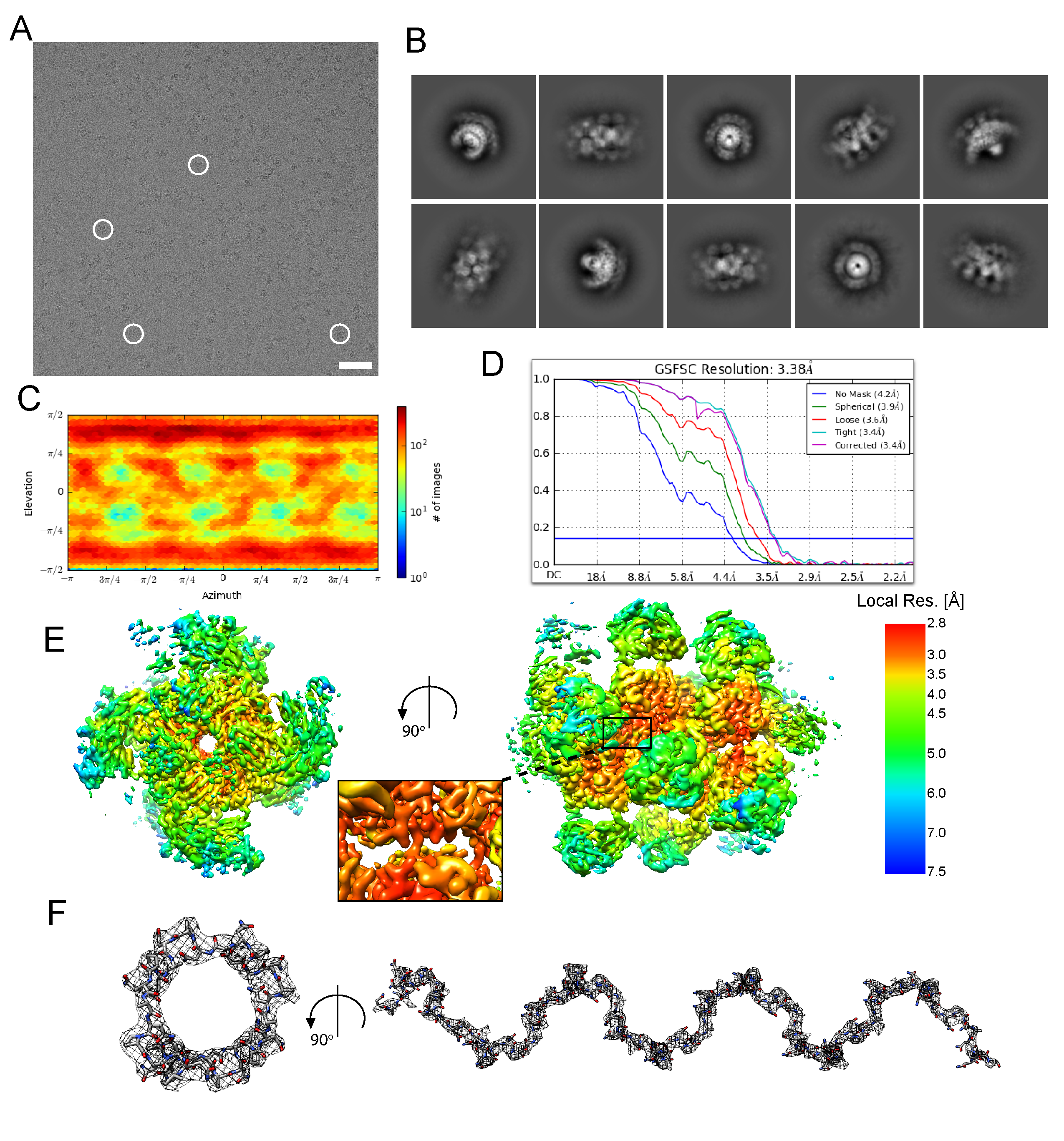

Supplement: S10 Fig — (A) Left panel–a representative cryoEM micrograph of the 850 Fab-PfCSP complex from a 200 kV screening microscope with individual particles highlighted with white circles. Scale bar: 50 nm. (B) Selected 2D class averages of the 850 Fab-PfCSP complex. (C) Particle orientation distribution plot. (D) Fourier shell correlation curve from the final 3D non-uniform refinement of the 850 Fab-PfCSP complex in cryoSPARC v2. (E) Local resolution (Å) plotted on the surface of the cryoEM map. (F) CryoEM map of PfCSP (grey mesh) with the model shown as sticks (black carbons). (TIF) [file ppat.1010999.s010.tif]

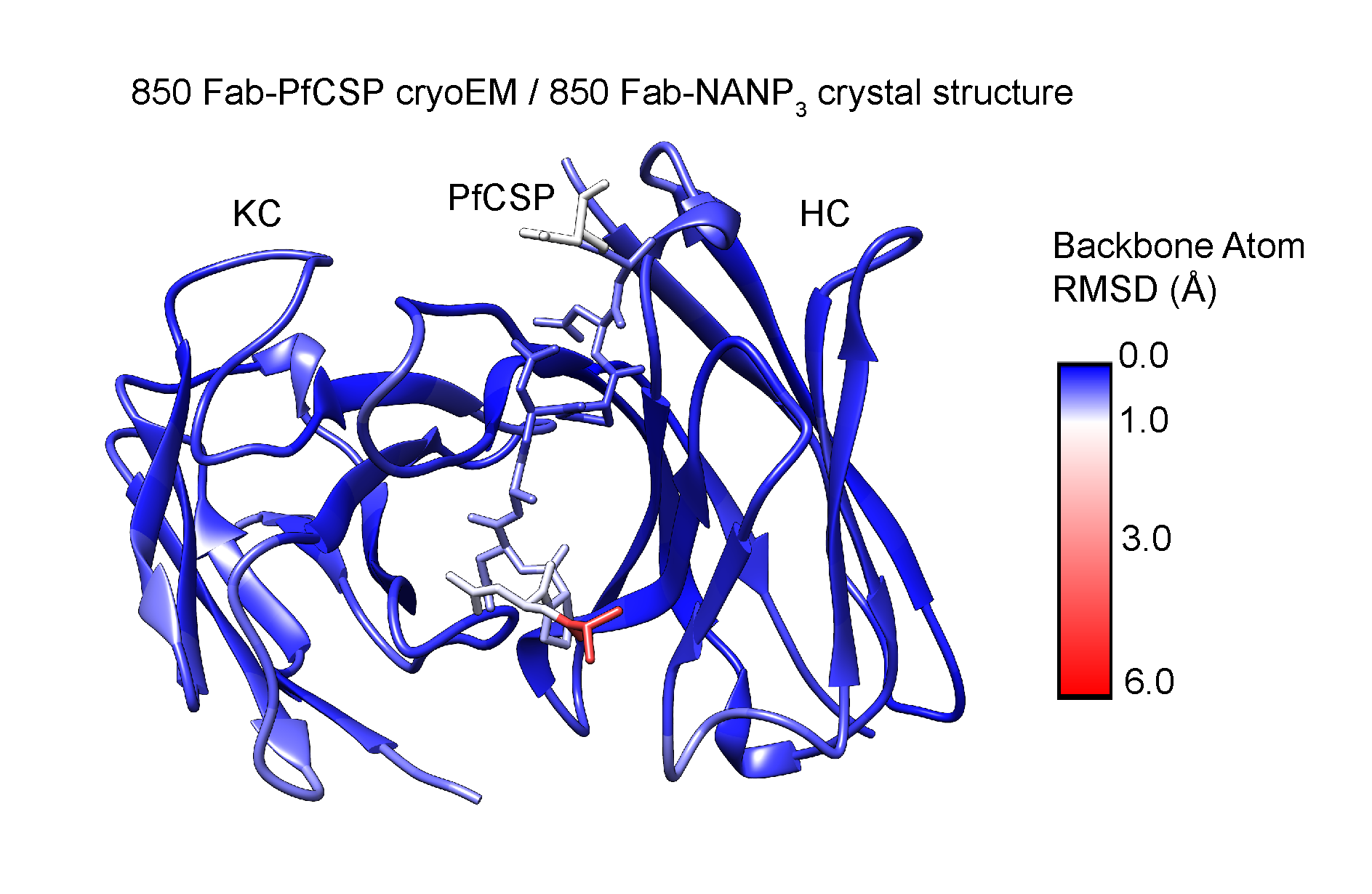

Supplement: S11 Fig — Models were aligned using PyMOL [61]. RMSD values were calculated using UCSF Chimera [57] and plotted by color on the secondary structure of the 850 Fab-PfCSP cryoEM structure. (TIF) [file ppat.1010999.s011.tif]

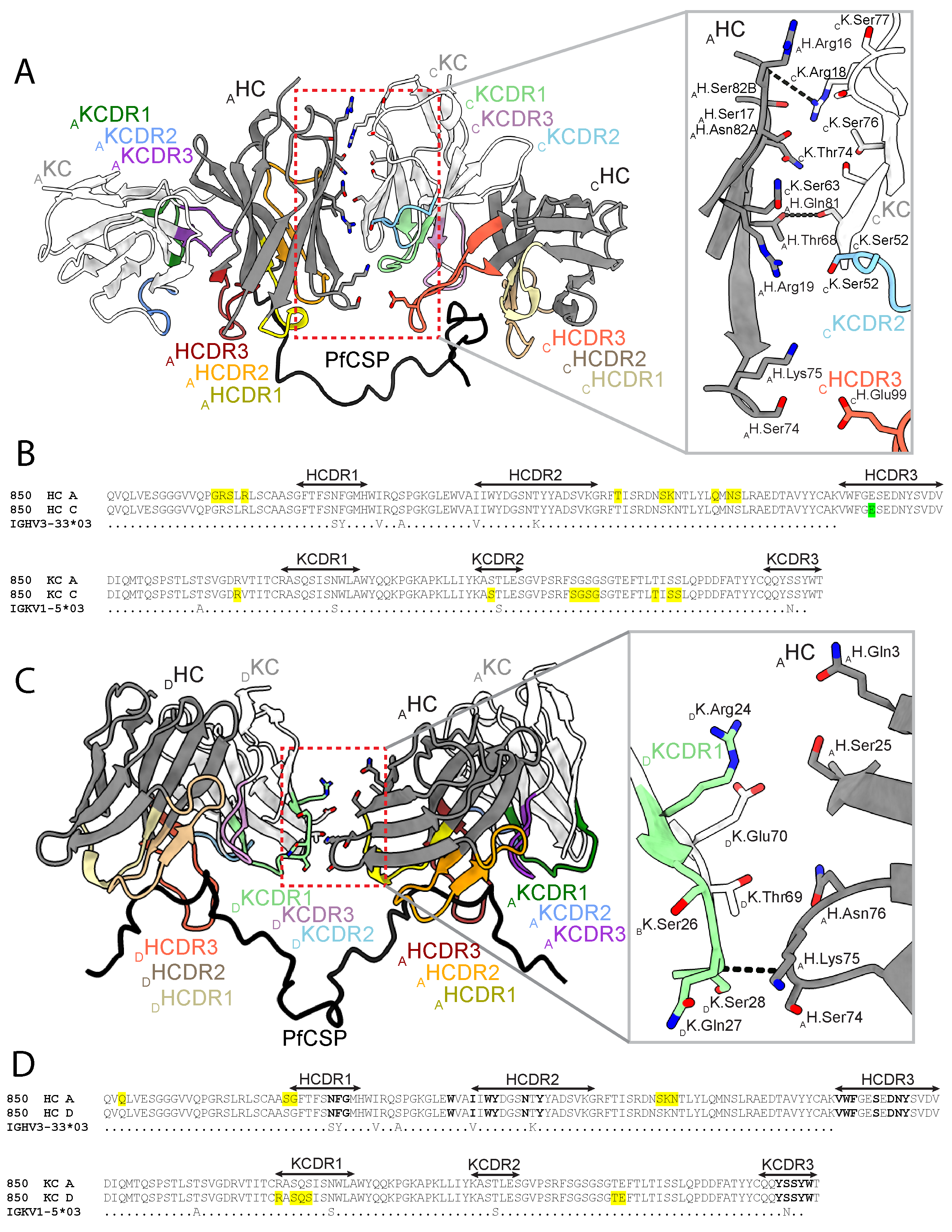

Supplement: S12 Fig — (A) Interface between Fabs A and C includes FR1 and FR3 of Fab A HC and FR1 and FR3 of Fab C KC, as well as two residues in CDR regions (K.Ser52 from KCDR2 and H.Glu99 from HCDR3 of Fab C). H-bonds are represented by dash lines. (B) Amino acid sequence alignment of mAbs 850 with the germline VH (top) and VK (bottom) Ig gene segments. Yellow highlight: germline-encoded residues involved in homotypic interactions; green highlight: somatically hypermutated residues involved in homotypic interactions that do not form interactions with PfCSP. (C) Interface between Fabs A and D. Black dashed line denotes H-bond. Residues forming Fab-Fab contacts are labeled with the position of the Fab (A and D) indicated in subscript. (D) Amino acid sequence alignment of mAbs 850 with the germline VH (top) and VK (bottom) Ig gene segments. Yellow highlight: germline-encoded residues involved in homotypic interactions. (TIF) [file ppat.1010999.s012.tif]

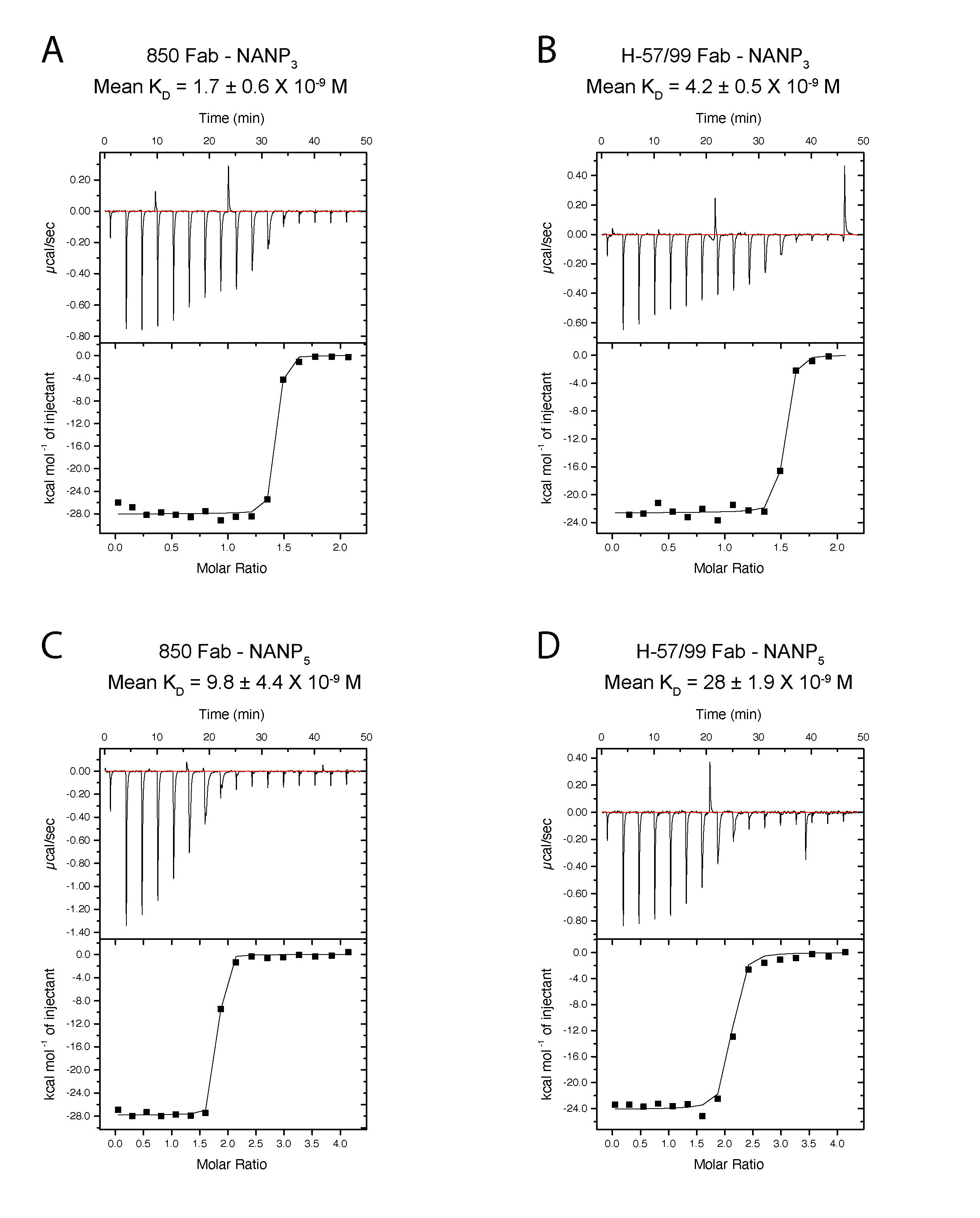

Supplement: S13 Fig — ITC measurements of 850 Fab and H-57/99 Fab binding to NANP3 (A and B, respectively), and 850 Fab and H-57/99 Fab binding to NANP5 (C and D, respectively) at 25°C. Representative raw data are shown above the corresponding plot and trendline of heat of injectant. Mean KD values resulting from at least duplicate experiments are indicated with the associated standard error of the mean (SEM). Stoichiometry (N) values were consistently found to be greater for NANP5 than NANP3 but are not reported due to large variability associated with measurement of peptide concentrations. (TIF) [file ppat.1010999.s013.tif]
