# Supplementary material for: High-density binding to Plasmodium falciparum circumsporozoite protein repeats by inhibitory antibody elicited in mouse with human immunoglobulin repertoire
Source: PLoS Pathog. 2022 Nov 28;18(11):e1010999. doi: 10.1371/journal.ppat.1010999 (PMC9762590; doi:10.1371/journal.ppat.1010999)
Supplement: S1 Table — (DOCX) [file ppat.1010999.s015.docx]

| **immunogen** | **mAb** | ***IGHV*** | ***IGKV*** |
| --- | --- | --- | --- |
| NANP_5.5_–1210 Fab–LS–Th2R | 850 | IGHV3-33 | IGKV1-5 |
| NANP_5.5_–1210 Fab–LS–Th2R | 851 | IGHV6-1 | IGLV9-49 |
| NANP_5.5_–ferritin–TSR | 831 | IGHV4-39 | IGKV1-33 |
| NANP_5.5_–ferritin–TSR | 855 | IGHV3-33 | IGLV1-40 |
| NANP_5.5_–ferritin–TSR | 861 | IGHV3-33 | IGKV1-5 |
| NANP_5.5_– LS–Th2R | 838 | IGHV3-33 | IGKV1-5 |
| NANP_5.5_– LS–Th2R | 841 | IGHV3-33 | IGKV1-5 |
| NANP_5.5_– LS–Th2R | 843 | IGHV3-33 | IGKV1-5 |
| NANP_5.5_– LS–Th2R | 844 | IGHV3-33 | IGKV1-5 |
| NANP_5.5_– LS–Th2R | 845 | IGHV3-33 | IGKV1-5 |
| NANP_5.5_– LS–Th2R | 846 | IGHV3-33 | IGKV1-5 |
| NANP_5.5_– LS–Th2R | 848 | IGHV3-33 | IGKV1-5 |
| NANP_5.5_– LS–Th2R | 849 | IGHV3-33 | IGKV1-5 |
| NANP_5.5_– LS–Th2R | 856 | IGHV3-33 | IGKV3D-15 |
| NANP_5.5_– LS–Th2R | 857 | IGHV3-33 | IGKV1-5 |
| NANP_5.5_– LS–Th2R | 858 | IGHV3-33 | IGKV3-20 |
| NANP_5.5_– LS–Th2R | 859 | IGHV3-33 | IGKV1-5 |
| NANP_5.5_– LS–Th2R | 860 | IGHV3-33 | IGKV1-5 |
| PfCSP | 839 | IGHV3-33 | IGKV1-5 |
| PfCSP | 840 | IGHV3-33 | IGKV1-5 |
| PfCSP | 847 | IGHV1-18 | IGKV1-13 |
