# Supplementary material for: High-density binding to Plasmodium falciparum circumsporozoite protein repeats by inhibitory antibody elicited in mouse with human immunoglobulin repertoire
Source: PLoS Pathog. 2022 Nov 28;18(11):e1010999. doi: 10.1371/journal.ppat.1010999 (PMC9762590; doi:10.1371/journal.ppat.1010999)
Supplement: S2 Table — (DOCX) [file ppat.1010999.s016.docx]

| **Peptide** | **Sequence** | **Experiment** |
| --- | --- | --- |
| NANP_3_ | NANPNANPNANP | Crystallization, ITC |
| NANP_5_ | NANPNANPNANPNANPNANP | ELISA, Negative Stain EM, ITC |
| NPNA_3_ | NPNANPNANPNA | SPR |
| NANP_6_ | NANPNANPNANPNANPNANPNANP | SPR |
| NVDPNANPNVDPNANPNVDP | NVDPNANPNVDPNANPNVDP | SPR |
| NPDPNANPNVDPNANP | NPDPNANPNVDPNANP | SPR |
| KQPADGNPDPNANPN | KQPADGNPDPNANPN | SPR |
