# Supplementary material for: High-density binding to Plasmodium falciparum circumsporozoite protein repeats by inhibitory antibody elicited in mouse with human immunoglobulin repertoire
Source: PLoS Pathog. 2022 Nov 28;18(11):e1010999. doi: 10.1371/journal.ppat.1010999 (PMC9762590; doi:10.1371/journal.ppat.1010999)
Supplement: S3 Table — (DOCX) [file ppat.1010999.s017.docx]

|  | **Antigen (BSA Å^2^)** | | **Interaction** | **850-HC** | **850-KC** |
| --- | --- | --- | --- | --- | --- |
|  |  | **Ala2 (19)** | vdW | Tyr58 |  |
|  |  | **Asn3 (20)** | vdW | Trp52, Tyr58, Asn100C |  |
|  |  | **Pro4 (126)** | vdW | Ile50, Trp52, Tyr58 | Tyr94, Trp95 |
|  |  | **Asn5 (135)** | vdW | Asp100B, Asn100C, Tyr100D | Tyr91, Ser92, Ser93, Tyr94, Trp95 |
|  |  | Asn^O^ | HB | Tyr100D^N^ |  |
|  |  | Asn^O^ | HB | Asn100C^ND2^ |  |
|  |  | Asn^OD1^ | HB |  | Tyr94^N^ |
|  |  | Asn^ND2^ | HB |  | Tyr91^O^ |
|  |  | Asn^ND2^ | HB |  | Tyr94^O^ |
|  |  | **Ala6 (17)** | vdW | Trp52, Asp100B, Asn100C, Tyr100D |  |
|  |  | **Asn7 (64)** | vdW | Trp52, Ser100, Tyr100D |  |
|  |  | Asn^O^ | HB | Trp52^NE1^ |  |
|  |  | **Pro8 (119)** | vdW | Phe32, Gly33, Ile50, Ile51, Trp52, Tyr52A, Val95, Tyr100D |  |
|  |  | Pro^O^ | HB | Tyr52A^N^ |  |
|  |  | Pro^O^ | HB | Gly33^N^ |  |
|  |  | **Asn9 (100)** | vdW | Asn31, Phe32, Gly33, Tyr52A, Val95, Trp96, Phe97, Tyr100D |  |
|  |  | Asn^OD1^ | HB | Gly33^N^ |  |
|  |  | Asn^ND2^ | HB | Trp96^O^ |  |
|  |  | **Ala10 (75)** | vdW | Asn31, Tyr52A |  |
|  |  | Ala^N^ | HB | Asn31^O^ |  |
|  |  | **Asn11 (13)** | vdW | Tyr52A |  |
|  |  |  |  |  |  |
|  |  | 11 |  |  |  |
| **H-bonds** |  | 689 |  |  |  |
| **Total BSA (Å^2^)** |  |  |  |  |  |
|  |  |  |  |  |  |

vdW: van der Waals interaction (5.0 Å cut-off)

HB: hydrogen bond (4.0 Å cut-off)
