# Supplementary material for: High-density binding to Plasmodium falciparum circumsporozoite protein repeats by inhibitory antibody elicited in mouse with human immunoglobulin repertoire
Source: PLoS Pathog. 2022 Nov 28;18(11):e1010999. doi: 10.1371/journal.ppat.1010999 (PMC9762590; doi:10.1371/journal.ppat.1010999)
Supplement: S4 Table — (DOCX) [file ppat.1010999.s018.docx]

|  | **Antigen (BSA Å^2^)** | | **Interaction** | **850-HC** | **850-KC** |
| --- | --- | --- | --- | --- | --- |
|  |  | **Ala66 (21)** | vdW | Tyr58 |  |
|  |  | Ala^O^ | HB | Tyr58^OH^ |  |
|  |  | **Asn67 (24)** | vdW | Asn100C |  |
|  |  | Asn^ND2^ | HB | Asn100C^OD1^ |  |
|  |  | **Pro68 (99)** | vdW | Ile50, Trp52, Tyr58 | Tyr94, Trp95 |
|  |  | **Asn69 (133)** | vdW | Asp100B, Asn100C, Tyr100D | Tyr91, Ser92, Ser93, Tyr94, Trp95 |
|  |  | Asn^O^ | HB | Tyr100D^N^ |  |
|  |  | Asn^OD1^ | HB |  | Tyr94^N^ |
|  |  | Asn^ND2^ | HB |  | Tyr91^O^ |
|  |  | Asn^ND2^ | HB |  | Tyr94^O^ |
|  |  | **Ala70 (30)** | vdW | Trp52, Asp100B, Tyr100D |  |
|  |  | **Asn71 (68)** | vdW | Trp52, Glu99, Ser100, Tyr100D |  |
|  |  | Asn^O^ | HB | Trp52^NE1^ |  |
|  |  | **Pro72 (119)** | vdW | Phe32, Gly33, Ile50, Ile51, Trp52, Tyr52A, Val95, Tyr100D |  |
|  |  | Pro^O^ | HB | Tyr52A^N^ |  |
|  |  | Pro^O^ | HB | Gly33^N^ |  |
|  |  | **Asn73 (112)** | vdW | Phe32, Gly33, Tyr52A, Val95, Trp96, Phe97, Gly98, Tyr100D |  |
|  |  | Asn^OD1^ | HB | Gly33^N^ |  |
|  |  | Asn^ND2^ | HB | Trp96^O^ |  |
|  |  | **Ala74 (66)** | vdW | Asn31, Tyr52A, Phe97 |  |
|  |  | Ala^N^ | HB | Asn31^O^ |  |
|  |  | **Asn75 (6)** | vdW | Tyr52A |  |
|  |  | **Pro76 (24)** | vdW | Phe97 |  |
|  |  |  |  |  |  |
| **H-bonds** |  | 12 |  |  |  |
| **Total BSA (Å^2^)** |  | 703 |  |  |  |
|  |  |  |  |  |  |

vdW: van der Waals interaction (5.0 Å cut-off)

HB: hydrogen bond (4.0 Å cut-off)
