# Supplementary material for: High-density binding to Plasmodium falciparum circumsporozoite protein repeats by inhibitory antibody elicited in mouse with human immunoglobulin repertoire
Source: PLoS Pathog. 2022 Nov 28;18(11):e1010999. doi: 10.1371/journal.ppat.1010999 (PMC9762590; doi:10.1371/journal.ppat.1010999)
Supplement: S5 Table — (DOCX) [file ppat.1010999.s019.docx]

|  | **Interface 1 (BSA Å^2^)** | | | | **Interface 2 (BSA Å^2^)** | | | | **Interface 3 (BSA Å^2^)** | | | |
| --- | --- | --- | --- | --- | --- | --- | --- | --- | --- | --- | --- | --- |
|  | **Fab A HC** | **Fab A KC** | **Fab B HC** | **Fab B KC** | **Fab A HC** | **Fab A KC** | **Fab C HC** | **Fab C KC** | **Fab A HC** | **Fab A KC** | **Fab D HC** | **Fab D KC** |
|  | Thr28 (17) |  | Ser55 (2) | Gln27 (40) | Gly15 (18) (HB) |  | Glu99 (12) | Arg18 (79) (HB) | Gln3 (2) |  |  | Arg24 (16) |
|  | Ser30 (9) |  | Asn56 (28) | Ser28 (5) | Arg16 (34) |  |  | Ser52 (20) | Ser25 (32) |  |  | Ser26 (27) |
|  | Asn31 (63) |  | Thr57 (17) | Ser93 (19) | Ser17 (24) |  |  | Ser63 (20) | Gly26 (6) |  |  | Gln27 (32) |
|  | Phe97 (55) |  | Tyr58 (34) | Tyr 94 (38) | Arg (20) |  |  | Gly64 (7) | Ser74 (48) (HB) |  |  | Ser28 (26) (HB) |
|  | Gly98 (2) |  |  |  | Thr68 (29) (HB) |  |  | Ser65 (45) (HB) | Lys75 (54) |  |  | Thr69 (36) |
|  | Glu99 (47) |  |  |  | Ser74 (3) |  |  | Gly66 (8) | Asn76 (18) |  |  | Glu70 (24) |
|  |  |  |  |  | Lys75 (9) |  |  | Thr74 (13) |  |  |  |  |
|  |  |  |  |  | Gln81 (25) |  |  | Ser76 (28) |  |  |  |  |
|  |  |  |  |  | Asn82A (38) |  |  | Ser77 (14) |  |  |  |  |
|  |  |  |  |  | Ser82B (22) |  |  |  |  |  |  |  |
| **Total BSA (Å^2^):** | 193 | 0 | 80 | 102 | 222 | 0 | 12 | 235 | 161 | 0 | 0 | 160 |
|  |  |  |  |  |  |  |  |  |  |  |  |  |

vdW: van der Waals interaction (5.0 Å cut-off)

HB: hydrogen bond (4.0 Å cut-off)
